# Comparative Chloroplast Genomics and Phylogenetic Analysis of *Zygophyllum* (Zygophyllaceae) of China

**DOI:** 10.3389/fpls.2021.723622

**Published:** 2021-09-24

**Authors:** Ling Zhang, Shu Wang, Chun Su, AJ Harris, Liang Zhao, Na Su, Jun-Ru Wang, Lei Duan, Zhao-Yang Chang

**Affiliations:** ^1^College of Life Science, Northwest A&F University, Yangling, China; ^2^Herbarium of Northwest A&F University, Yangling, China; ^3^College of Life Science, Tarim University, Alar, China; ^4^Key Laboratory of Biological Resource Protection and Utilization of Tarim Basin Xinjiang Production & Construction Group, Alar, China; ^5^Guangdong Provincial Key Laboratory of Applied Botany, South China Botanical Garden, Chinese Academy of Sciences, Guangzhou, China

**Keywords:** chloroplast genome, comparative analysis, phylogeny, *Zygophyllum*, Zygophyllaceae

## Abstract

The genus *Zygophyllum* comprises over 150 species within the plant family Zygophyllaceae. These species predominantly grow in arid and semiarid areas, and about 20 occur in northwestern China. In this study, we sampled 24 individuals of *Zygophyllum* representing 15 species and sequenced their complete chloroplast (cp) genomes. For comparison, we also sequenced cp genomes of two species of *Peganum* from China representing the closely allied family, Nitrariaceae. The 24 cp genomes of *Zygophyllum* were smaller and ranged in size from 104,221 to 106,286 bp, each containing a large single-copy (LSC) region (79,245–80,439 bp), a small single-copy (SSC) region (16,285–17,146 bp), and a pair of inverted repeat (IR) regions (3,792–4,466 bp). These cp genomes contained 111–112 genes each, including 74–75 protein-coding genes (PCGs), four ribosomal RNA genes, and 33 transfer RNA genes, and all cp genomes showed similar gene order, content, and structure. The cp genomes of *Zygophyllum* appeared to lose some genes such as *ndh* genes and rRNA genes, of which four rRNA genes were in the SSC region, not in the IR regions. However, the SC and IR regions had greater similarity within *Zygophyllum* than between the genus and *Peganum*. We detected nine highly variable intergenic spacers: *matK-trnQ, psaC-rps15, psbZ-trnG, rps7-trnL, rps15-trnN, trnE-trnT, trnL-rpl32, trnQ-psbK*, and *trnS-trnG*. Additionally, we identified 156 simple sequence repeat (cpSSR) markers shared among the genomes of the 24 *Zygophyllum* samples and seven cpSSRs that were unique to the species of *Zygophyllum*. These markers may be useful in future studies on genetic diversity and relationships of *Zygophyllum* and closely related taxa. Using the sequenced cp genomes, we reconstructed a phylogeny that strongly supported the division of Chinese *Zygophyllum* into herbaceous and shrubby clades. We utilized our phylogenetic results along with prior morphological studies to address several remaining taxonomic questions within *Zygophyllum*. Specifically, we found that *Zygophyllum kaschgaricum* is included within *Zygophyllum xanthoxylon* supporting the present treatment of the former genus *Sarcozygium* as a subgenus within *Zygophyllum*. Our results provide a foundation for future research on the genetic resources of *Zygophyllum*.

## Introduction

*Zygophyllum* was originally described by Linnaeus (1753) and is one of the 25 genera comprising the plant family Zygophyllaceae according to the Angiosperm Phylogeny Group (APG) IV (2016). This genus consists of approximately 150 species (Beier et al., 2003), which are widely distributed in northern and southern Africa, the Mediterranean region, and central Asia and Australia (White, 1983; Zyl and Marias, 1999; Retief, 2000). Among the Asian species, ca. 20 occur in northwestern China, where all species of *Zygophyllum* grow preferentially on sandy gravel terraces, hilly slopes, and gravel dunes and pediments, and where *Zygophyllum xanthoxylon* (Bunge) Maxim. is a dominant species in the desert and semidesert areas (Zeng et al., 2004). Species of *Zygophyllum* are highly adapted to dry conditions and provide critical ecosystem services within arid environments of Xinjiang, Inner Mongolia, and Gansu in China.

Within the family Zygophyllaceae, generic circumscriptions were first established by Sheahan and Chase (1996) by using the molecular phylogenetic analysis of the chloroplast (cp) marker, *rbcL*, and Sheahan and Chase (2000) later delimited the subfamily Zygophylloideae based on *trnL-F* and *rbcL*. A comprehensive phylogenetic analysis of *trnL* and morphological data for 43 species of Zygophylloideae showed that the genera, *Augea* (monotypic, southern Africa), *Tetraena* (monotypic, China), and *Fagonia* (ca. 30 species), are nested within *Zygophyllum* (ca. 150 species) (Beier et al., 2003). When *Zygophyllum* includes these genera, its monophyly is highly supported (Beier et al., 2003). In later studies, molecular phylogenies were used for divergence time dating and biogeographic analyses revealing that Asian *Zygophyllum* colonized the Asian interior from Africa in the early Oligocene, began diversification in the early Miocene, and underwent rapid radiation in the late Miocene (Wu et al., 2015, 2018).

Similar to the family as a complete, *Zygophyllum* of China has undergone considerable taxonomic change, especially from ca. 1980 to 2011. During this time, several new species were described and added to *Zygophyllum*, although some of these have not been widely accepted as truly distinctive (Liou, 1980, 1998; Zhao and Zhu, 2003; Liou and Zhou, 2008; Yang, 2011). Simultaneously, *Sarcozygium* Bunge, which contains one or two species and occurs in Xinjiang as well as Kazakhstan and Mongolia, was accepted as an independent genus (Liou, 1998; Yang, 2011), but in the Flora of China, it was merged to *Zygophyllum* (Liou and Zhou, 2008). Within *Zygophyllum*, species are typically delimited morphologically based especially on differences in the number of leaflets per leaf (1, 2–5 pairs), relative length of the stamens compared to the petals (equal, longer, or shorter), and features of the capsule (winged or not, ridged or not, and dehiscent or indehiscent) (Beier et al., 2003; Liou and Zhou, 2008). For example, Z*ygophyllum kaschgaricum* Boriss. has a capsule that is linearly ovoid, spindle-like, or obovoid, and smaller than that of *Z. xanthoxylon*, whose capsule is nearly spherical. Similarly, *Zygophyllum latifolium* Schrenk has leaflets that are rotund to oblong, but the leaflets of *Zygophyllum rosowii* Bunge are ovate. However, these morphological characters alone are often insufficient to distinguish Chinese species due to more or less continuous variation. Thus, for Chinese species, a clear set of morphological characters to support the identification of plants in the field is needed. Moreover, species boundaries remain poorly resolved for the Chinese members of the genus, in part because they have been sparsely sampled in prior molecular phylogenetic studies and due to the lack of existing genetic materials.

Among Chinese *Zygophyllum*, complete cp genomes have been published for *Z. xanthoxylon* and *Zygophyllum fabago* L. (Xu et al., 2020), but additional cp genomes are needed to represent the genus in China and to support resolving species boundaries and phylogenetic relationships. The cp genomes have been shown to be a valuable tool for resolving species relationships and boundaries within a molecular phylogenetic framework (Mao et al., 2014; Shaw et al., 2014; Hollingsworth et al., 2016; Fu et al., 2017; Duan et al., 2020) and are also regarded as an important source for simple sequence repeat (cpSSR) markers that can be utilized in the studies of barcoding and population genetics (Powell et al., 1996; Wu et al., 2010; Perdereau et al., 2014; Park et al., 2016; Mohammad-Panah et al., 2017). The cp genome in most angiosperms is relatively small in size, maternally inherited, and highly conserved with low nucleotide substitution rates except within the extremely mutable intergenic spacer regions (Birky et al., 1983; Bendich, 2004; Moore et al., 2007; Liu et al., 2019). Thus, cp genomes are relatively easy to sequence due to their small size, and the phylogenetic analysis and interpretation are fairly straightforward due to the ease of alignment among conserved regions and lack of genetic anomalies associated with biparental inheritance, respectively. Moreover, obtaining the sequences of complete cp genomes is increasingly fast and cheap with the continual development of new sequencing technology. As a consequence, more and more complete cp genomes have been generated to resolve taxonomic and phylogenetic controversies.

Increasingly, the comparative analyses of the complete cp genome structures among closely related species have also yielded a wealth of information for taxonomically or phylogenetically recalcitrant lineages (Hu et al., 2017), such as *Orychophragmus* (Hu et al., 2015, 2016), *Epimedium* (Zhang et al., 2016), and *Orchids* (Niu et al., 2017). Broadly, the structures of cp genomes in angiosperms are highly conserved; they are circular double-helices consisting of a large single-copy (LSC) region, a small single-copy (SSC) region, and a pair of inverted repeat (IR) regions and are the sizes of cp genomes range from 100 to 220 kb in length (Ozeki et al., 1989; Marcelo et al., 2015). However, IRs expand and contract (Goulding et al., 1996; Downie and Jansen, 2015; He et al., 2017; Ye et al., 2018), yielding informative differences in plastome size and/or gene order. Additionally, cp gene gain and loss and pseudogenization are widespread within plastomes of angiosperms (Jansen et al., 2008; Delannoy et al., 2011; Quan and Zhou, 2011). Therefore, the structural analysis of plastomes is an additional, valuable tool to aid in species delimitation.

In this study, we sequenced 24 complete cp genomes of Chinese species of *Zygophyllum* representing 15 species to elucidate species boundaries and phylogenetic relationships. For additional comparison, we also included cp genomes of two species of *Peganum*, which were previously separated from Zygophyllaceae and are presently treated within Nitrariaceae (Angiosperm Phylogeny Group (APG) IV, 2016). The main objectives of this study were to (1) compare the sequences, structures, and gene organization of cp genomes within Chinese *Zygophyllum* with additional comparisons to representative *Peganum*; (2) detect highly variable regions and cpSSRs for the future development of genetic markers for *Zygophyllum*; and (3) integrate molecular phylogenetic evidence from the cp genomes with morphology for the delimitation of species of *Zygophyllum* in China.

## Materials and Methods

### Sample Preparation and DNA Sequencing

We collected fresh, healthy leaves from 24 individuals representing 15 species of *Zygophyllum* comprising six samples of *Z. rosowii*, two of *Z. kaschgaricum*, three each of *Z. xanthoxylon* and *Z. fabago*, and one each of the remaining 10 species. We collected all fresh samples in the field and stored them in silica sand. The density of our sampling within species reflected the levels of continuous variation that we observed in the field and ongoing taxonomic disputes. Specifically, for *Z. rosowii* and *Z. fabago*, we observed considerable intraspecific variation but relative uniformity across species, while *Z. kaschgaricum* and *Z. xanthoxylon* have poorly resolved boundaries. We selected and sequenced *Peganum harmala* L. and *Peganum nigellastrum* Bunge to represent the outgroup. *Peganum* belongs to Nitrariaceae now but was formerly treated within Zygophyllaceae (Liou, 1998). Following collections in the field, we stored the silica-dried leaves in a 4°C refrigerator until processing. In the field, we also collected voucher specimens, which were deposited in the herbarium of the Northwest A&F University (WUK) (Table 1).

**Table 1:** Details of sampled accessions used in this study.

**No**	**Samples**	**Origin, Voucher**	**Latitude**	**Longitude**	**Altitude (m)**	**District**	**Morphologic Observation**
1	*Z. fabago*	L. Zhang et al. ZL011 (WUK)	43°33′	87°53′	1,122	Xinjiang Urumqi	Flower/seed
2	*Z. fabago*_a	Z.Y.Chang et al. 2019230 (WUK)	47°9′	87°33′	442	Xinjiang Fuhai	
3	*Z. fabago*_b	Z.Y.Chang et al. 2019231 (WUK)	47°9′	87°33′	442	Xinjiang Fuhai	Flower/seed
4	*Z. gobicum*	Z.Y.Chang et al. 2019247 (WUK)	39°21′	100°3′	1,327	Gansu Zhangye	Flower
5	*Z. jaxarticum*	Z.Y.Chang et al. 2019316 (WUK)	44°9′	85°36′	830	Xinjiang Shawan	Flower
6	*Z. kaschgaricum*_b	L. Zhang et al. ZL073 (WUK)	42°47'	86°16'	1,050	Xinjiang Korla	
7	*Z. kaschgaricum*_h	L. Zhang et al. ZL076 (WUK)	43°11'	90°43'	885	Xinjiang Hami	Flower/seed
8	*Z. loczyi*	Z.Y.Chang et al. 2019055 (WUK)	41°46'	102°58'	1,009	Inner Mongolia Alxa Zuoqi	Flower
9	*Z. macropodum*	L. Zhang et al. ZL017 (WUK)	40°59′	105°56′	1,002	Xinjiang Urumqi	Flower/seed
10	*Z. macropterum*	Zhang L et al. ZL015 (WUK)	43°38′	87°53′	1,496	Xinjiang Urumqi	Flower/seed
11	*Z. mucronatum*	Z.Y.Chang et al. 2019033 (WUK)	38°13′	106°21′	1,153	Inner Mongolia Lingwu	Flower
12	*Z. neglectum*	Zhang L et al. 2019246 (WUK)	45°59′	90°8′	991	Xinjiang Qinhai	Seed
13	*Z. obliquum*	L. Zhang et al. ZL072 (WUK)	37°11'	75°18'	4,200	Xinjiang Tashkurgan	Seed
14	*Z. potaninii*	L. Zhang et al. ZL078 (WUK)	42°23'	95°26'	1,353	Xinjiang Hami	Flower/seed
15	*Z. pterocarpum*	Z.Y.Chang et al. 2019047 (WUK)	40°59′	105°56′	1,259	Inner Mongolia Wulatehouqi	Flower/seed
16	*Z. rosowii*	Z.Y.Chang et al. 2019050 (WUK)	40°59′	105°56′	1,259	Inner Mongolia Wulatehouqi	Flower/seed
17	*Z. rosowii_*a	Z.Y.Chang et al. 2019302 (WUK)	38°58′	75°27′	1,845	Xinjiang Aketedu	
18	*Z. rosowii*_b	L. Zhang et al. ZL077 (WUK)	43°29′	90°59′	1,968	Xinjiang Hami	
19	*Z. rosowii*_c	Z.Y.Chang et al. 2019053 (WUK)	40°48′	104°28′	1,376	Inner Mongolia Alxa Zuoqi	Flower/seed
20	*Z. rosowii*_d	Z.Y.Chang et al. 2019065 (WUK)	41°46'	102°58'	1,009	Inner Mongolia Alxa Zuoqi	
21	*Z. sinkiangense*	Z.Y.Chang et al. 2019263 (WUK)	42°29′	86°15′	1,321	Xinjiang Korla	Seed
22	*Z. xanthoxylon*_b	L. Zhang et al. ZL074 (WUK)	42°47'	86°16'	1,050	Xinjiang Korla	Flower/seed
23	*Z. xanthoxylon*_g	Z.Y.Chang et al. 2019059 (WUK)	41°34′	96°58′	1,944	Gansu Jiuquan	
24	*Z. xanthoxylon*_n	L. Zhang et al. ZL075 (WUK)	39°52′	106°45′	1,040	Inner Mongolia Ordos	
25	*Peganum harmala*	Z.Y.Chang et al. 2019304 (WUK)	36°12′	107°39′	1,164	Gansu Qincheng	
26	*Peganum nigellastrum*	Z.Y.Chang et al. 2019009 (WUK)	36°48′	107°8′	1,305	Gansu Huanxian	

Using the silica-dried samples, we performed extractions of complete genomic DNA *via* a modified cetyltrimethylammonium bromide (CTAB) method (Doyle and Doyle, 1987) and assessed the quality and concentration of the extracted DNA with agarose gel electrophoresis and a NanoDrop 2000 spectrophotometer (Thermo Fisher Scientific, USA). The high-quality DNAs served as material to construct a library for sequencing on an Illumina NovaSeq 6000 system following the instructions of the manufacturer. The libraries consisted of fragments with an average of 270 bp and sequencing comprised 150 bp paired-end reads.

### Genome Assembly, Annotation, and Sequence Analyses

Sequencing yielded a total of 205,535,340 bp of raw reads, which we cleaned and subsequently assembled in NOVOPlasty 2.7.2 (Dierckxsens et al., 2017) using the *rbcL* gene sequence of *Z. rosowii* (JF944812) as the seed and the complete cp genome sequence of *Tetraena mongolica* Maxim. (MH325021) as the reference based on the nested position of *Tetraena* (monotypic) within *Zygophyllum* (Beier et al., 2003) and the availability of the sequence. We annotated all protein-coding genes (PCGs), transfer RNAs (tRNAs), and ribosomal RNAs (rRNAs) of the cp genomes of *Zygophyllum* and *Peganum* using GeSeq (Tillich et al., 2017) on the Chlorobox web server (https://chlorobox.mpimp-golm.mpg.de/index.html) and PGA (Qu et al., 2019). We compared annotations from the two sources and made final adjustments manually in Geneious version 11.0.2 (Zufall, 2009). We detected the boundaries of the IR regions using Repeat Finder (Benson, 1999) implemented in Geneious 11.0.2, and we visualized the cp genomic maps with OGDRAW (Lohse et al., 2013) *via* Chlorobox under default settings with manual quality control. Using MAFFT version 7 (Katoh and Standley, 2013), we aligned the complete cp genomes of *Zygophyllum* and *Peganum* and then performed manual editing in Geneious version 11.0.2. Finally, we submitted all final cp genome sequences generated in this study to the NCBI database with the accession number (Table 1).

### Comparison of cp Genomes and Identification of Polymorphic Regions

Based on our annotations, we compared the IR/SC boundary regions of the 15 species of *Zygophyllum* to each other, to other representatives of Zygophyllaceae [*Teteaena mongolica* Maxim. (subfamily Zygophylloideae), *Guaiacum angustifolium* Engelm. (subfamily Larreoideae), *Tribulus terrestris* L. (subfamily Balanitoideae)], and to *P. harmala*. Moreover, we more deeply compared the cp genomes among the 15 species of *Zygophyllum* using mVISTA in LAGAN mode (Frazer et al., 2004) with our newly annotated sequence of *Z. fabago* as the reference.

For determining the levels of polymorphism of sites within the cp genomes, we conducted a sliding window analysis of the 24 aligned plastomes of *Zygophyllum* in DnaSP version 5.10.01 (Librado and Rozas, 2009), and we determined nucleotide variability (Pi) using a step size of 200 bp and a window length of 600 bp. We performed the sliding window analysis for the complete cp genomes and for the LSC, SSC, and IR regions individually. We also detected and analyzed the distributions of cpSSRs using MISA (Beier et al., 2017) with the minimum number of repeats set to eight for mononucleotide repeats, six for dinucleotides, five for trinucleotides, four for tetranucleotides, three for pentanucletides, and three for hexanucletides. We defined SSRs as comprising one to six repetitive DNA bases, and we verified all repeats manually.

### Phylogenetic Analyses

We reconstructed a phylogeny using all 24 complete cp genomes of *Zygophyllum* and two *Peganum* complete cp genomes sequenced in this study as well as four additional publicly available sequences representing the family that we downloaded from the NCBI (Supplementary Table 1). The additional representatives comprised *Teteaena mongolica* (MH325021), *Larrea tridentata* (DC.) Coville (KT272174.1), *G. angustifolium* (MK726011.1), and *Teteaena terrestris* (NC_046758). For phylogenetic analyses, we generated six datasets in Geneious version 11.0.2 (Zufall, 2009) consisting of the complete cp genomes and extracted sequences representing all coding sequences (CDSs); the LSC, the SSC, and the IR regions; and non-coding sequences (NCSs) and performed new alignments using MAFFT version 7 under default parameters with subsequent manual adjustments (Katoh and Standley, 2013).

Our phylogenetic analyses consisted of maximum likelihood (ML) and Bayesian inference (BI). ML analyses were implemented using RAxML-HPC BlackBox version 8.1.24 (Stamatakis, 2014) on the CIPRES Science Gateway (http://www.phylo.org/) (Miller et al., 2010) under the GTRGMMA+I model with 1,000 bootstrap replicates to determine branch support. For BI, we determined that GTR+I+G was the best fitting model of evolution for the complete cp genome, CDS, NCS, and LSC datasets based on jModelTest version 2.1.7 (Darriba et al., 2012). Trees based on the SSC and IRs were poorly based on the ML analyses (as described in the “Results” section), such that we did not perform BI. We performed BI using two independent runs in MrBayes version 3.2 (Ronquist et al., 2012) consisting of 10,000,000 generations each with sampling every 1,000 generations and the first 25% of trees discarded as burn-in. From the remaining trees, we determined posterior probability (PP) values after using Tracer version 1.7 (Rambaut et al., 2018) to verify the stationarity (ESS > 200) and convergence. We visualized the final ML and BI trees in Figtree version 1.4.3 (http://tree.bio.ed.ac.uk/software/figtree/).

### Microscopy Observation of Flower and Seed

For morphological and microscopic analysis, we sampled mature fruits and/or flowers in cases where they were available from the same specimens used for DNA extraction (Table 1). We fixed the flower samples in FAA solution (formalin/acetic acid/70% ethanol = 10:5:85) after collection and stored the dry fruits in paper bags at 4°C. We performed dissections of the flowers using tweezers and dissecting needles and observed them under a Nikon SMZ25 stereomicroscope. We also took photographs of flowers that were still attached to branches using a Nikon D5100 digital camera. For the fruits, we peeled away the pericarp and washed the seeds two times in an ultrasonic washer (2 min/time). Thereafter, we took photographs of the seeds when dry and wet. Using the available specimens, we were able to sample 10 flowers and 50 seeds for each unique species of *Zygophyllum* represented in this study.

## Results

### General Features of cp Genomes Sampled in This Study

Each of the complete cp genomes from 24 samples of *Zygophyllum* and two samples of *Peganum* yielded ca. 2G of the clean sequence data. Within *Zygophyllum*, the complete cp genomes ranged in size from 104,211 bp for *Zygophyllum potaninii* Maxim. to 106,286 bp for *Zygophyllum sinkiangense* Y. X. Liou, and the GC content had a narrow range from 33.7 to 34.1% (Table 2). All cp genomes in *Zygophyllum* included the LSC region of 79,245 to 80,439 bp, the SSC region of 16,285 to 17,146 bp, and the IR regions of 3,792 to 4,466 bp. The complete cp genome sequences of *Peganum* were 159,956 and 160,066 bp. All cp genomes sequenced in this study were deposited in GenBank (MW307829–MW771517) (Table 2).

**Table 2:** Summary statistics for complete chloroplast genomes of the sampled accessions of *Zygophyllum* and *Peganum*.

**Samples**	**Total length**	**LSC** **length**	**SSC length**	**IR** **length**	**Coding sequences(bp)**	**Non-coding sequences(bp)**	**Total genes**	**Protein** **coding genes**	**tRNA**	**rRNA**	**GC%**	**Accession numbers**
*Z. fabago*	104,477	79,245	16,754	4,239	47,462	57,015	112	75	33	4	34	MW417249
*Z. fabago_a*	104,489	79,317	16,690	4,241	47,405	57,084	112	75	33	4	34	MW551564
*Z. fabago_b*	104,601	79,354	16,777	4,235	47,402	57,199	112	75	33	4	33.9	MW417250
*Z. gobicum*	105,233	79,785	16,842	4,303	47,447	57,786	112	75	33	4	33.8	MW489449
*Z. jaxarticum*	104,447	79,390	16,495	4,281	47,408	57,039	112	75	33	4	33.9	MW387263
*Z. kaschgaricum_b*	105,398	80,005	16,971	4,211	47,668	57,730	112	75	33	4	34	MW417253
*Z. kaschgaricum*_h	105,430	80,034	16,974	4,211	47,722	57,708	112	75	33	4	34	MW417256
*Z. loczyi*	105,529	79,979	16,886	4,332	47,510	58,019	112	75	33	4	33.8	MW489448
*Z. macropodum*	105,443	79,975	16,890	4,289	47,384	58,059	112	75	33	4	33.9	MW551563
*Z. macropterum*	105,145	80,230	16,285	4,315	47,627	57518	112	75	33	4	33.9	MW307829
*Z. mucronatum*	104,740	79,685	16,405	4,325	47,483	57,257	112	75	33	4	33.9	MW387266
*Z. neglectum*	104,998	80,050	16,426	4,261	47,486	57,512	112	75	33	4	33.9	MW387264
*Z. obliquum*	105,650	80,366	17,050	4,117	47,684	57,966	111	74	33	4	33.8	MW525443
*Z. potaninii*	104,211	79,481	17,146	3,792	47,609	56,602	112	75	33	4	33.9	MW771516
*Z. pterocarpum*	105,062	79,704	16,594	4,382	47,612	57,450	112	75	33	4	33.9	MW387265
*Z. rosowii*	104,535	79342	16551	4,321	47,362	57,173	112	75	33	4	33.8	MW525444
*Z. rosowii*_a	104,640	79,480	1,522	4,319	47,319	57,321	112	75	33	4	33.8	MW387262
*Z. rosowii_b*	104,739	79,575	16,530	4,317	47,453	57,286	112	75	33	4	33.8	MW557319
*Z. rosowii_c*	104,913	79,676	16,957	4,321	47396	57,517	112	75	33	4	33.8	MW771517
*Z. rosowii_d*	104,758	79,551	16587	4,310	47,321	57,437	112	75	33	4	33.8	MW417251
*Z. sinkiangense*	106,286	80,439	16915	4466	47,504	58,782	112	75	33	4	33.7	MW557318
*Z. xanthoxylon*_b	105,427	80,019	16,986	4,211	47,950	57,477	112	75	33	4	34	MW417252
*Z. xanthoxylon*_g	105,319	79,948	16,949	4,211	47,719	57,600	112	75	33	4	34.1	MW417255
*Z. xanthoxylon*_n	105,361	79,972	16,967	4,211	47,722	57,639	112	75	33	4	34.1	MW417254
*P. harmala*	159,956	88,097	1,8759	26,550	71,261	88,695	133	90	35	8	37.5	MW477239
*P. nigellastrum*	160,066	88,274	18854	26,469	72,590	87,476	133	90	35	8	37.5	MW477240

We successfully, fully annotated all 26 cp genomes newly sequenced in this study using PGA. We found that the cp genomes of *Zygophyllum* contained a total of 111–112 genes, among which there were 33 tRNA genes, four rRNA genes, and 75 PCGs, except in *Zygophyllum obliquum* Popov., which lacked *ndhH* and, thus, had 74 PCGs (Table 2). In contrast, the cp genomes of the two species of *Peganum* contained 138 coding genes comprising 35 tRNA genes, eight rRNA genes, and 95 PCGs. The cp genomes of *Zygophyllum* possessed four categories of genes based on KOG (Tatusov et al., 2003): genes related to photosynthesis-, transcription- and translation-related genes, RNA genes, and other functionally unknown genes.

The *Zygophyllum* cp genome harbored 104 unique genes, consisting of 67 PCGs, 33 tRNA genes, and four rRNA genes (Table 3). The SSC region contained 10 PCGs (*ccsA, ndhD, ndhH, ndhI, psaC, rpl32, rps7, rps12, rps15*, and *ycf68*), six tRNAs (*trnA-UGC, trnI-GAU, trnL-UAG, trnN-GUU, trnR-ACG*, and *trnV-GAC*), and four rRNAs (*rrn4.5, rrn5, rrn16*, and *rrn23*), while the LSC region contained 58 PCGs and 21 tRNAs. The IR regions included four PCGs (*rpl2, rpl23, rps19*, and *ycf15*) and three tRNAs (*trnH-GUG, trnI-CAU*, and *trnL-CAA*). Within the SC regions, there were only three NADH dehydrogenase genes (*ndhI, ndhD*, and *ndhH*), and all were pseudogenized, while the IR regions did not contain any NADH dehydrogenase genes or any rRNAs. In the *P. harmala* cp genome, which is representative of both sequenced genomes of *Peganum*, we observed 133 genes consisting of 90 PCGs, 35 tRNA genes, and eight rRNA genes. The IR region in the *P. harmala* cp genome included 20 genes consisting of eight PCGs, six tRNAs, and four rRNA genes, and IR of *T. terrestris* and *G. angustifolium* contained 18 genes, while *T. mongolica* had seven genes. Thus, *T. mongolica* was more similar to *Zygophyllum* with respect to the gene content of the IR regions. Within the cp genomes of *Zygophyllum*, we identified 16 genes that harbored introns comprising eight PCGs and six tRNA genes (Table 3). Of the intron-containing genes, 14 possessed only 1 intron, while *clpP* and *ycf3* possessed 2 introns in both *Zygophyllum* and *Peganum*.

**Table 3:** Functional annotations of cp genes *Zygophyllum* based on KOG annotation. The *ndhH* gene is absent in *Z. obliquum*.

**Category of genes**	**Category of gene**	**Name of gene**
Photosynthesis-related genes	Rubisco	*rbcL*
	Photosystem I	*psaA, psaB, psaC, psaJ, PsaI*
	Photosystem II	*psbF, psbL, psbN, psbB, psbC, psbA, psbD, psbE, psbH, PsbZ, psbK, psbJ, PsbI, psbT, psbM*
	cytochrome b/f complex	petD^*^, petB^*^, petA, petG, petL, petN
	NADH dehydrogenase	*ndhI, ndhD, ndhH*
	ATP synthase	atpB, atpE, atpA, atpF^*^, atpH, atpl
Transcription- and translation-related genes	transcription	rpoA, rpoB, rpoC1^*^, rpoC2
	ribosomal proteins	rpl2* × 2, rpl14, rpl16^*^, rpl22, rpl23 × 2, rpl33, rpl32, rpl36, rpl20, rps2, rps3, rps4, rps7, rps8,rps11, rps12^*^, rps12,rps16^*^, rps18, rps14, rps15, rps19 × 2
RNA genes	ribosomal RNA	*rrn5, rrn4.5, rrn16, rrn23*
	transfer RNA	trnK-UUU^*^, trnI-GAU^*^, trnA-UGC^*^, trnG-UCC^*^, trnV-UAC^*^, trnL-UAA^*^, trnS-GCU, trnS-GGA, trnY-GUA, trnS-UGA, trnL-CAA × 2, trnL-UAG, trnR-ACG, trnD-GUC, trnE-UUC, trnfM-CAU, trnW-CCA, trnP-UGG, trnH-GUG × 2, trnI-CAU × 2, trnQ-UUG, trnT-UGU, trnF-GAA, trnM-CAU, trnR-UCU, trnT-GGU, trnC-GCA, trnG-GCC, trnN-GUU, trnV-GAC
Other genes	RNA processing	*matK*
	C-type cytochrome synthesis gene	*ccsA*
	Subunit of acetyl-CoA	*accD*
	Envelope membrane protein	*cemA*
	proteolysis	*clpP* ^**^
Genes with unknown function	hypothetical chloroplast reading frames	ycf3^**^, ycf4, ycf15 × 2, ycf68^*^

** *contains two introns, ×2 showed genes duplicated*.

### IR Boundary Analyses

**Figure 1:**
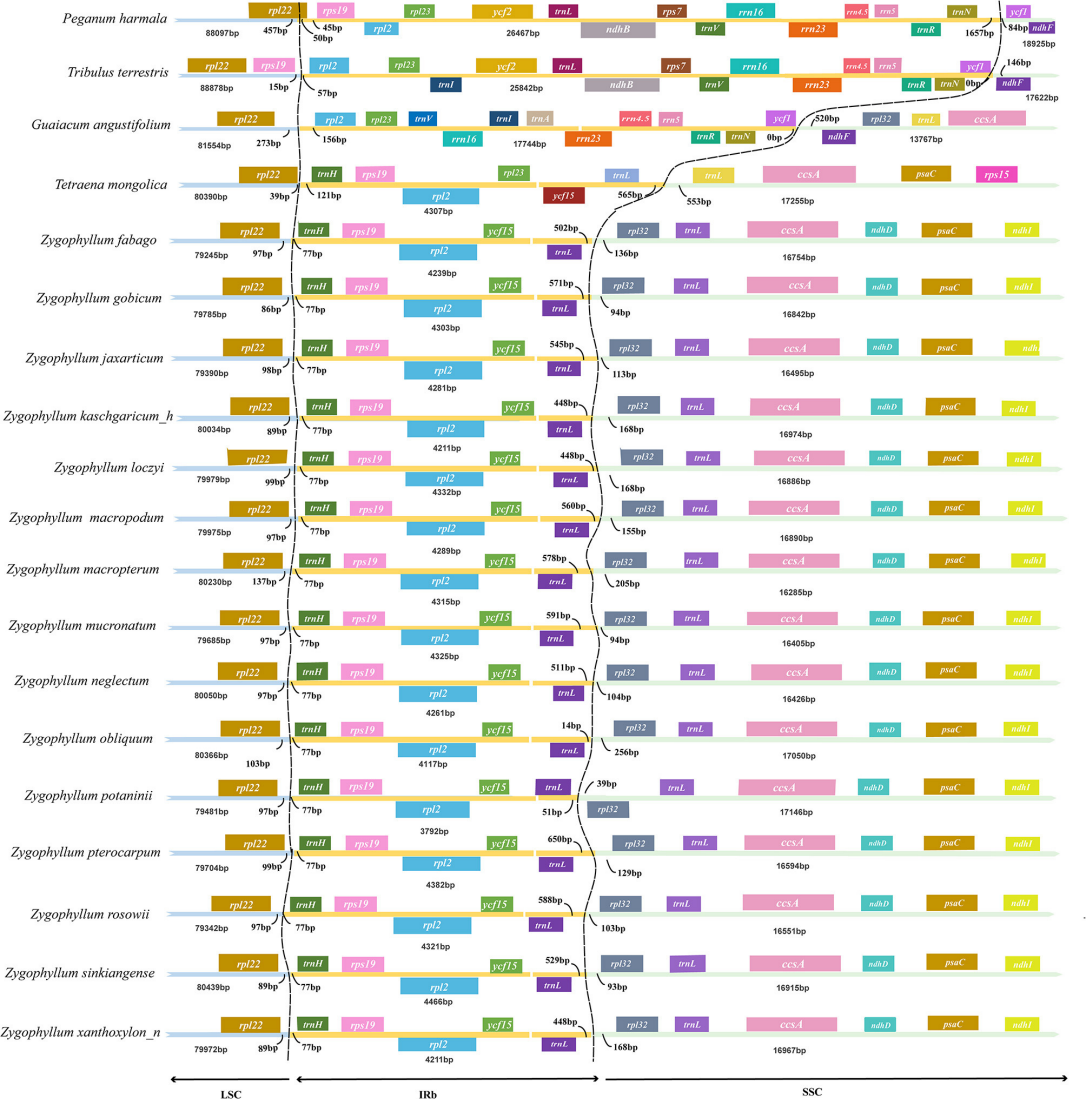
Comparison of large single-copy (LSC), small single-copy (SSC), and inverted repeat (IR) borders among plastomes of Zygophyllaceae.

We compared the IRs within *Zygophyllum* and to representative samples of three genera of Zygophyllaceae: *Tribulus, Larrea*, and *Tetraena*. We also summarized IRs of Zygophyllaceae to *P. harmala* of Nitrariaceae. The sizes of the IRs and the boundary locations were very similar within *Zygophyllum* and different among genera and families (Figure 1). Among *Zygophyllum* species, the boundary between the IR and LSC regions was placed in the intergenic region *rpl22*-*trnH*, with *rpl22* being 86–137 bp and *trnH* being 77 bp away from the IRb/LSC boundary. The IRb/SSC boundary in *Zygophyllum* occurred between *trnL-CAA* and *rpl32* with 14–588 bp from *trnL-CAA* to the IRb/SSC border and 39–256 bp from *rpl32* to the IRb/SSC border (Figure 1). The IR/SC boundary positions in *P. harmala, Teteaena terrestris*, and *G. angustifolium* were more similar but differed greatly from *Tetraena* and *Zygophyllum* in Zygophylloideae.

### Comparative cp Genomic Analysis

**Figure 2:**
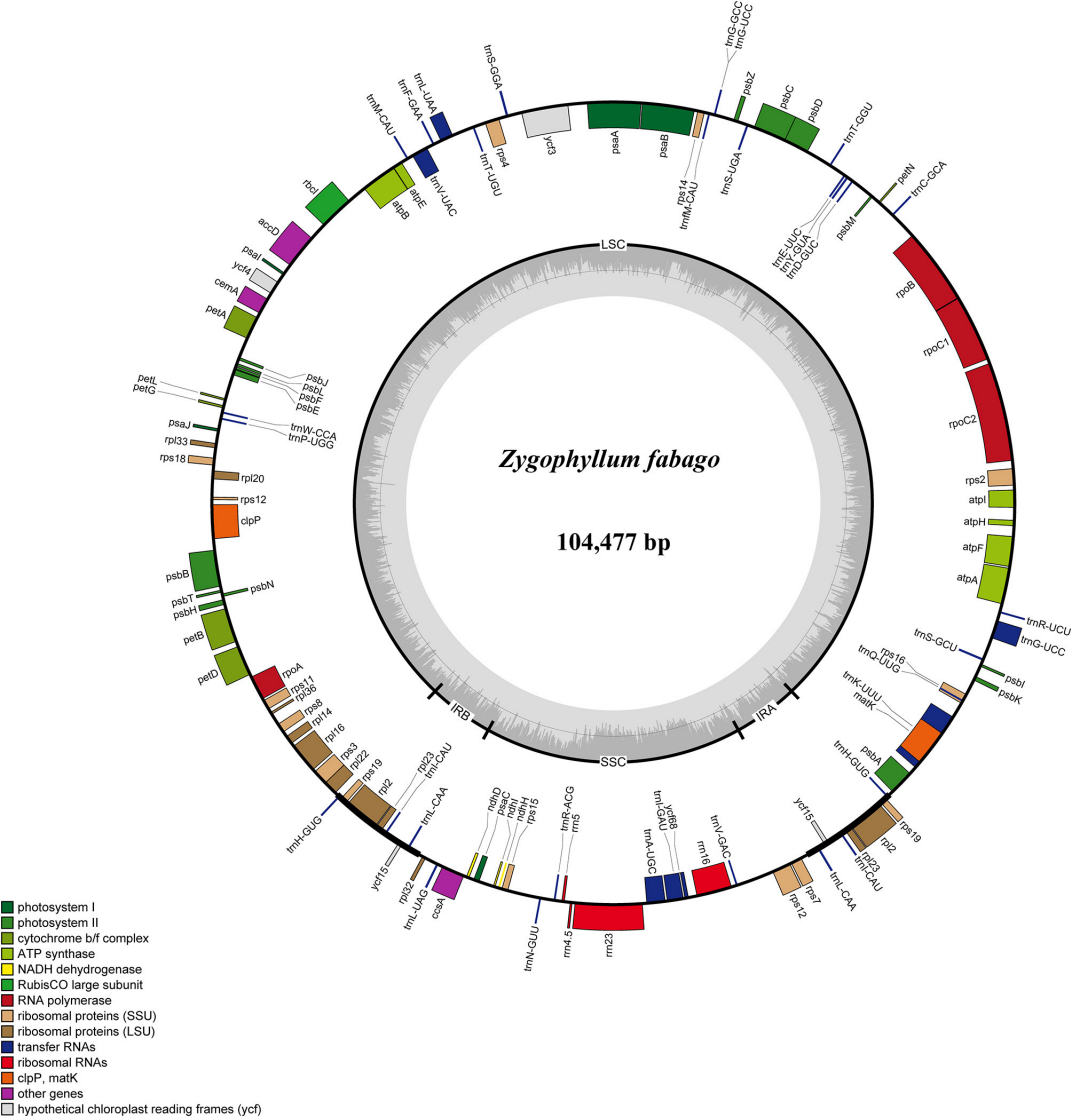
Gene map of the *Zygophyllum fabago* chloroplast (cp) genome. The genes inside the circle are transcribed clockwise, while those outside the circle are transcribed counterclockwise.

**Figure 3:**
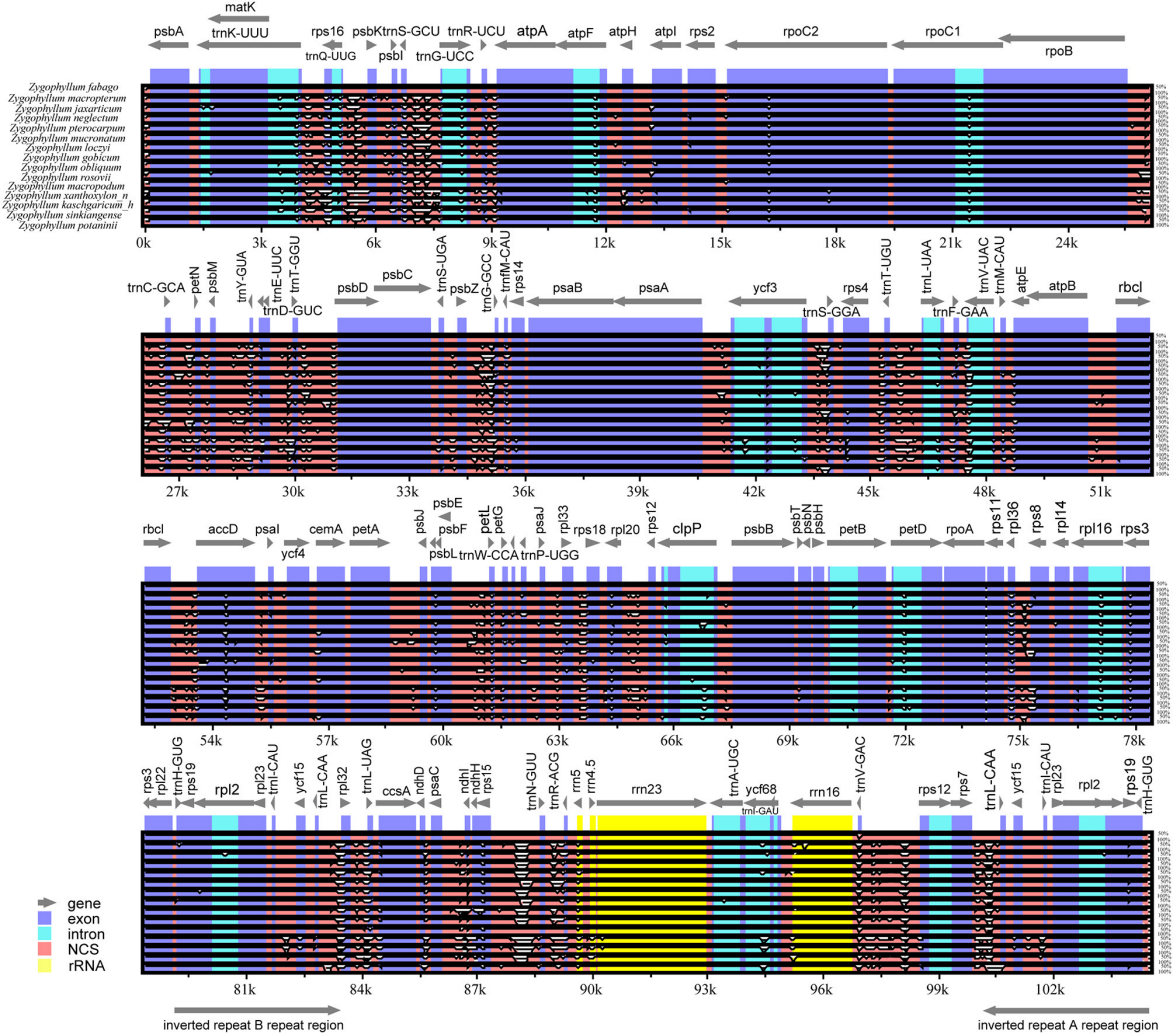
Comparison of plastomes of 15 species of *Zygophyllum* using *Z. fabago* as reference. Gray arrows and thick black lines above the alignment indicate gene orientation. Exons and introns are shown in blue (dark and light, respectively). Other intergenic spacers are shown in red. Ribosomal RNA genes are indicated in yellow. The *y*-axis indicates the percentage of identity, ranging from 50% to 100%.

We conducted comparisons of 15 cp genome sequences as representatives of Chinese *Zygophyllum* in mVISTA using our annotated genome of *Z. fabago* (Figure 2) as a reference to understand the distributions of conserved and divergent sequences among species (Figure 3). The comparative analysis revealed that NCSs were much more variable than CDSs. There was also considerable divergence among intergenic gene sequences (IGSs), such as *psbZ*-*trnG, trnK*-*rps16*, and *trnS*-*trnG* (Figure 3).

Furthermore, we examined the numbers of variable sites and nucleotide variability (Pi) across the cp genomes and among regions using DnaSP version 5.10.01 (Table 4). The results showed that the IRs were less divergent than those of the LSC and SSC regions. The number of variable sites was 5,331 across the cp genomes, 3,840 in the LSC region, 1,307 in the SSC region, and 130 in the IR regions (Table 4). The IR regions showed the lowest nucleotide diversity (Pi = 0.00948), while the SSC region had the highest (Pi = 0.01692).

**Table 4:** Analyses of variable sites in cp genomes of *Zygophyllum*.

	**Number of sites**	**Number of variable sites**	**Number of informative sites**	**Nucleotide diversity**
LSC region	88491	3840	2243	0.01229
SSC region	20348	1307	628	0.01692
IR region	4783	130	96	0.00948
Complete cp genome	118133	5331	3127	0.01306

### Highly Variable Regions and cpSSRs

**Figure 4:**
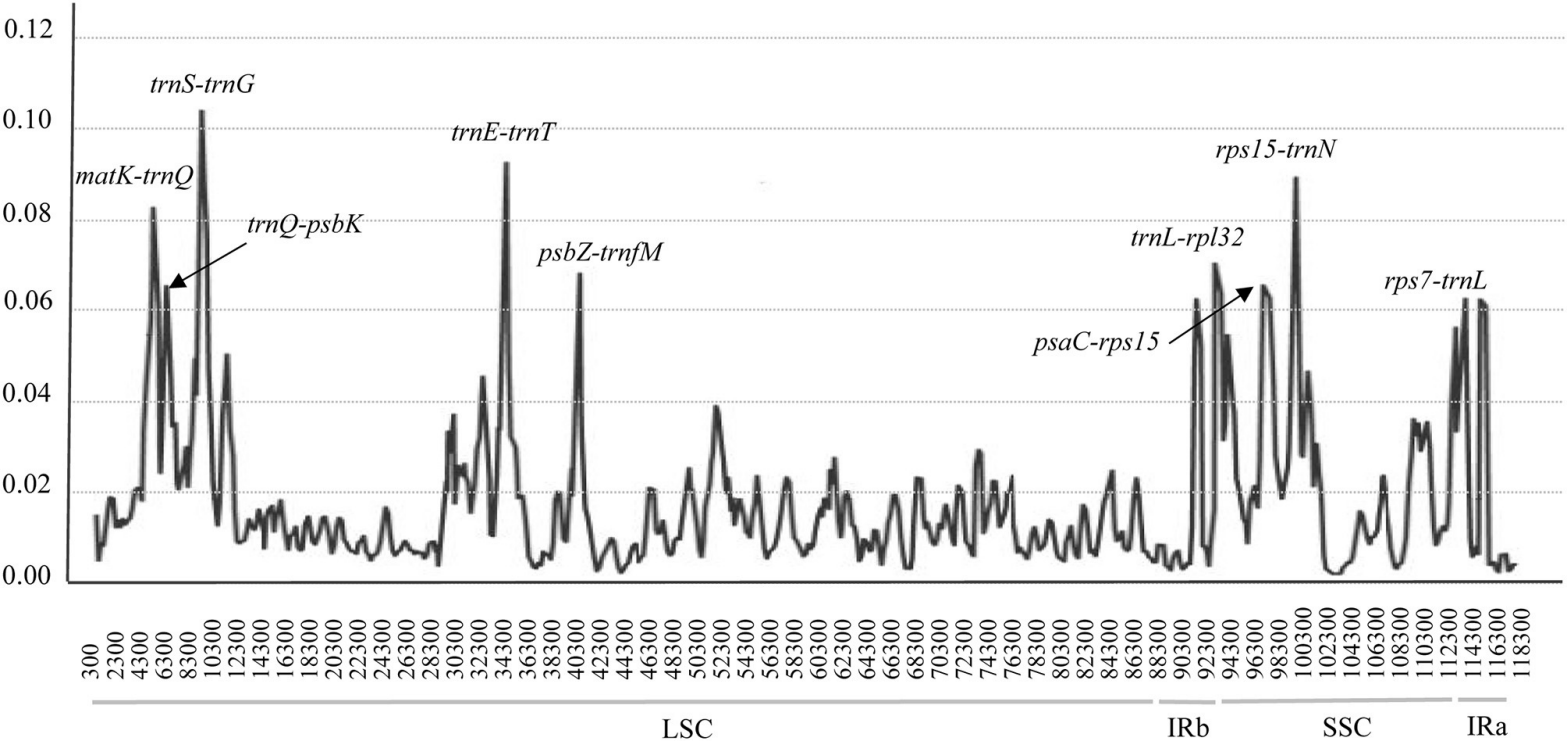
Hypervariable regions within cp genomes of species of *Zygophyllum* using a sliding window analysis. Window lengths: 600 bp; step size: 200 bp. Nine regions with the highest Pi values were marked out. LSC: large single-copy region, IR: inverted repeat region, SSC: small single-copy region. *x*-axis: position of the midpoint of a window; *y*-axis: nucleotide diversity of each window.

To detect highly variable regions, or hypervariable regions, among the 24 complete cp genomes of *Zygophyllum*, we conducted a sliding window analysis using DnaSP version 5.10.01. The sliding window analysis revealed nine highly variable regions with Pi ranging from 0.00063 to 0.10663 across 24 complete cp genomes (Figure 4). The highly variable regions comprised the intergenic spacer regions: *matK-trnQ, psaC-rps15, psbZ-trnG, rps7-trnL, rps15-trnN, trnE-trnT, trnL-rpl32, trnQ-psbK*, and *trnS-trnG*. Among the nine highly variable regions, five regions (*matK*-*trnQ, psbZ-trnG, trnE-trnT, trnQ-psbK*, and *trnS-trnG*) were located in the LSC, and four regions (*psaC-rps15, rps7-trnL, rps15-trnN*, and *trnL-rpl32*) were in the SSC. None of the hypervariable regions were in the IRs, which had more conserved sequences.

The nine hypervariable regions ranged from 917 bp (*trnL-rpl32)* to 2004 bp (*matK-trnQ*) in length (Table 5). There were 965 variable sites in the nine highly variable regions, including 545 parsimony informative sites. The Pi values of nine hypervariable variable sites ranged from 0.03495 to 0.08242 and were highest in *trnL-rpl32* and *trnS-trnG* (Table 5).

**Table 5:** Nine hypervariable regions of cp genomes of *Zygophyllum*.

**No**.	**region**	**Length (bp)**	**Variable sites**	**Parsimony informative sites**	**Nucleotide diversity (Pi)**
1	*matK-trnQ*	2,004	174	119	0.03854
2	*trnS-trnG*	1,621	104	66	0.05536
3	*trnQ-psbK*	1,445	81	50	0.03495
4	*trnE-trnT*	1,196	73	50	0.03794
5	*psbZ-trnG*	1103	54	33	0.04384
6	*trnL-rpl32*	917	58	42	0.08242
7	*psaC-rps15*	1,800	189	56	0.03879
8	*rps15-trnN*	1,619	132	92	0.03776
9	*rps7-trnL*	1609	100	37	0.0441
	Total	1,3314	965	545	0.04597

**Figure 5:**
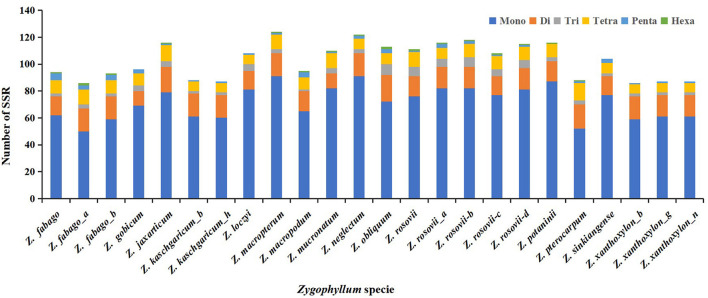
Number of simple sequence repeats (SSRs) from 24 *Zygophyllum* samples.

We detected and analyzed the occurrence, type, and distributions of SSRs of the 24 *Zygophyllum* complete cp genomes. In total, we found 156 cpSSRs, ranging from 86 cpSSRs in *Z. xanthoxylon*_*b, Z. xanthoxylon*_*g*, and *Z. fabago* to 124 cpSSRs in *Zygophyllum macropterum* (Figure 5, Supplementary Table 2). Among these cpSSR repeats, mononucleotide repeats (58–75%) and dinucleotide repeats (10–20%) were the most common, followed by tetranucleotide repeats (8–13%). Based on this, the mononucleotide repeats may be the most suitable targets for future studies of genetic diversity or species delimitation in the *Zygophyllum*. The proportions of trinucleotide, pentanucleotide, and hexanucleotide repeats were relatively low for each sample (Supplementary Table 2). Among mononucleotide repeats, polyA (20–38% of all SSRs) and polyT (35–48% of all SSRs) repeats were the most common, while polyC and polyG repeats were less frequent (Supplementary Table 2). For 156 cpSSRs, seven were shared across all 24 accessions of *Zygophyllum* sampled in this study (Supplementary Table 2).

**Figure 6:**
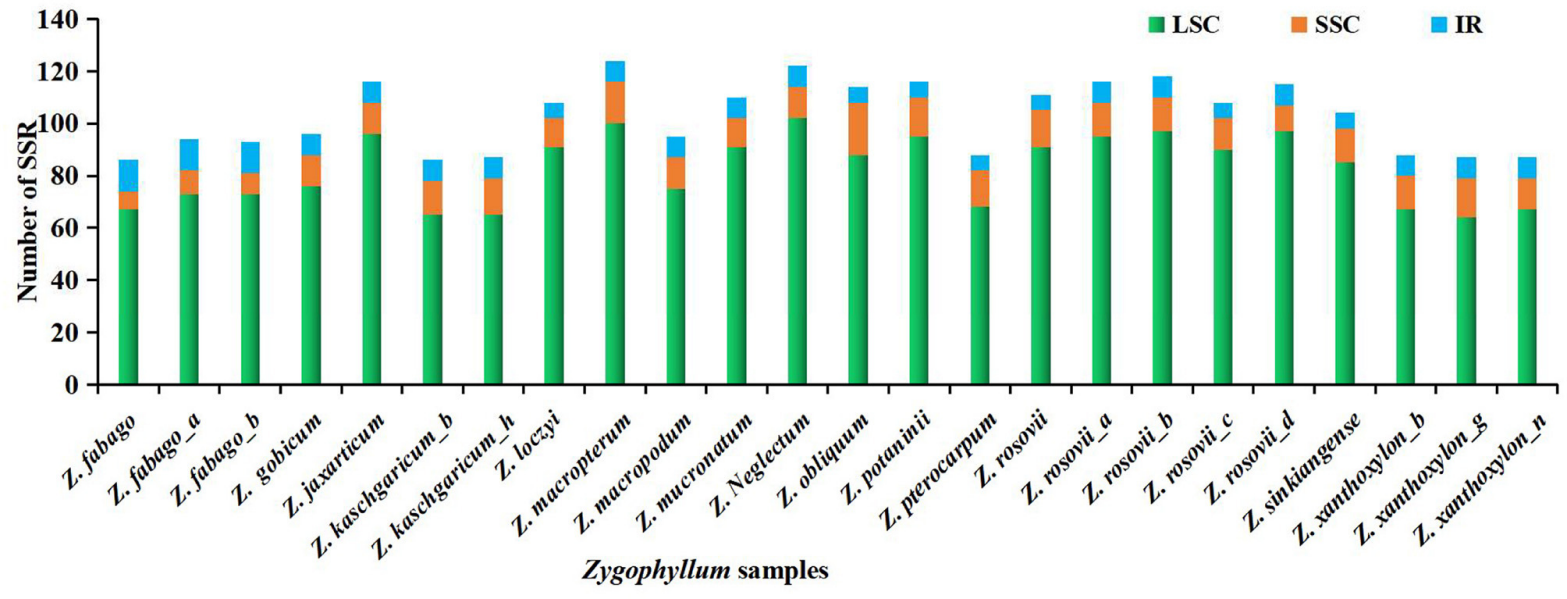
Number and distribution of SSRs in 24 complete cp genomes of *Zygophyllum* samples.

We found that SSRs were non-randomly distributed in the cp genomes of *Zygophyllum*. Of all cpSSRs, 74–84% were located within the LSC region, while only 8–18% and 5–14% were located within the SSC and IR regions, respectively (Figure 6, Supplementary Table 3). Similarly, most SSRs also occurred within the NCS regions (92–97%) compared to the CDS regions (3–8%) (Supplementary Table 3).

### Phylogenetic and Morphological Analyses

**Figure 7:**
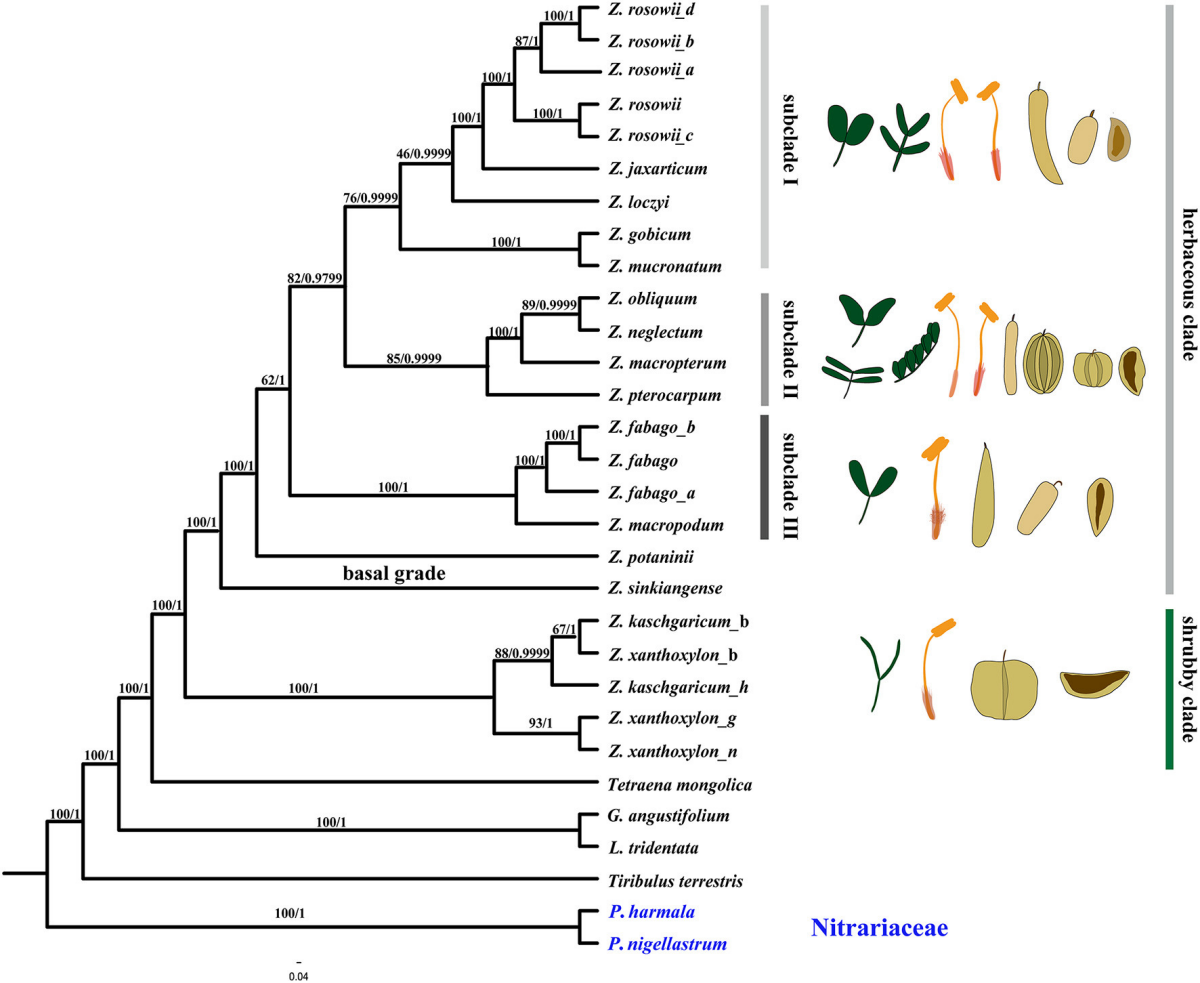
Phylogenetic relationships of *Zygophyllum* and its allies obtained from an ML and BI analysis of the complete cp genome. Numbers above the branches are the bootstrap values and Bayesian posterior probabilities, respectively.

To investigate evolutionary relationships between species of *Zygophyllum* in China and preliminarily assess species boundaries, we performed the phylogenetic analyses using ML and BI on the complete cp genomes as well as independently for all CDSs, all NCS regions, the LSC, the SSC, and IR regions. All trees resulting from the analyses of the complete cp genomes (Figure 7), CDSs (Supplementary Figure 1), NCSs, (Supplementary Figure 2), and LSCs (Supplementary Figure 4) showed similar topologies, except that the ML and BI trees of the NCSs differed slightly, with the ML tree being more similar to those obtained from the other datasets (Supplementary Figures 2, 3). Trees based on the SSC and IRs were poorly supported and were not further considered in this study (Supplementary Figures 5, 6).

The reconstructed phylogenies (Figure 7, Supplementary Figures 1–4) from the complete cp genome, the LSC, CDS, and NCS datasets showed that species of *Zygophyllum* clustered into two main clades (Figure 7), while the two *Peganum* species as the outgroup had a far phylogenetic relationship to *Zygophyllum*. One clade comprised *Z. kaschgaricum* and *Z. xanthoxylon* with high support [maximum likelihood bootstrap (MLBS) = 100%, PP = 1.0] and represents shrubby species that were formerly classified into *Sarcozygium* (Sheahan and Chase, 2000; Liou and Zhou, 2008). This clade was sister to the rest of the Chinese *Zygophyllum*, which is composed exclusively of herbaceous species. Within the shrubby clade, the accessions of *Z. kaschgaricum* and *Z. xanthoxylon* did not form mutually monophyletic groups (Figure 7).

**Figure 8:**
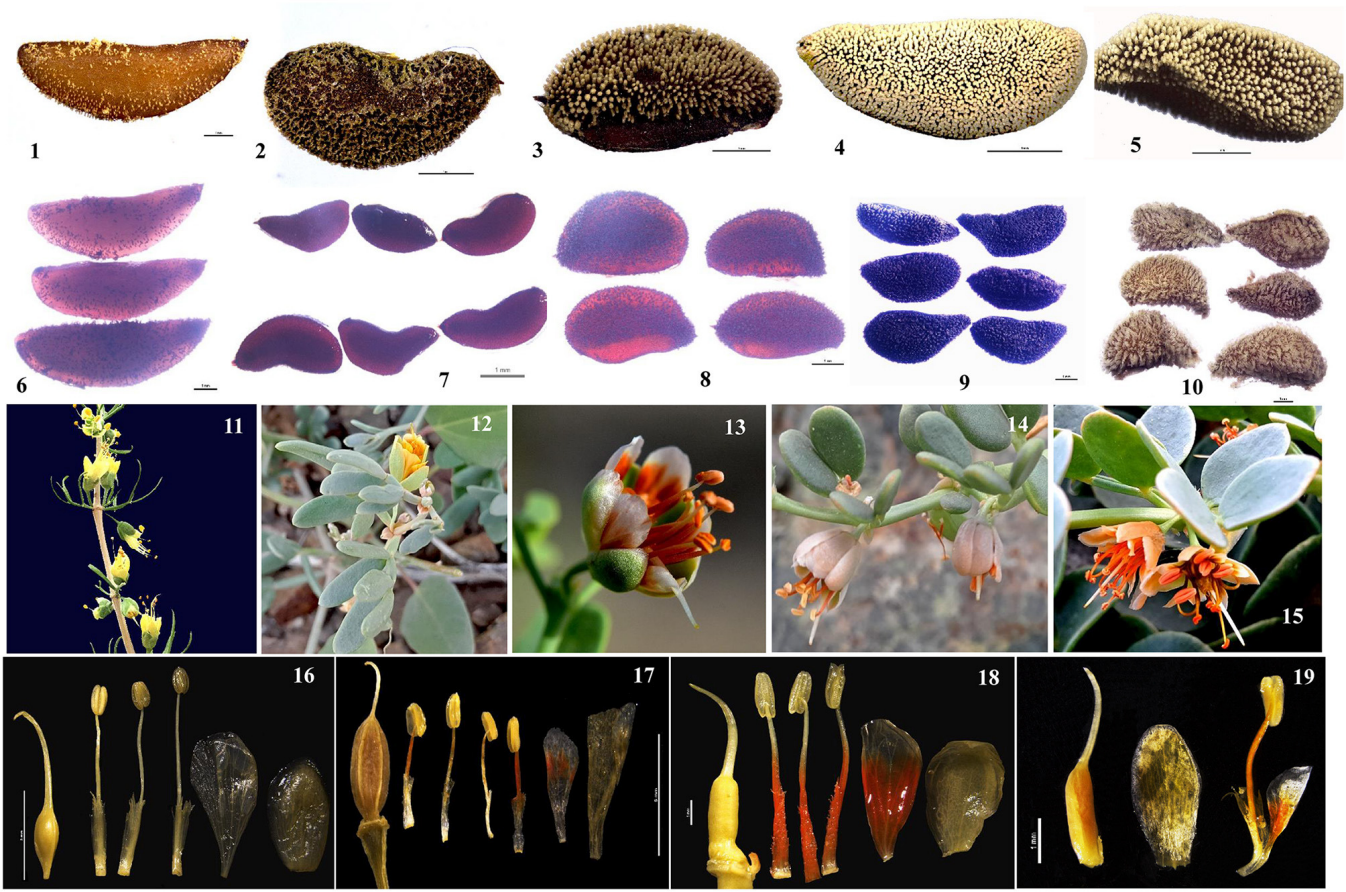
The morphological variation of species in *Zygophyllum*. Morphological characteristics of species of *Zygophyllum* representing the clade of woody shrubs (*Z. xanthoxylon_b*: 1, 6, 12, 17) and herbaceous species of the basal grade (*Z. potaninii:* 10,16; *Z. sinkiangense*: 11) and subclades I (*Z. rosowii*: 4, 8, 15, 20), II (*Z. pterocarpum*: 3, 9, 13, 18; *Z. neglectum:5*), and III (*Z. fabago*: 2, 7, 14, 19).

The herbaceous clade contained a basal grade and three distinct subclades. Subclade I comprised nine accessions representing *Zygophyllum gobicum, Zygophyllum jaxarticum, Zygophyllum loczyi, Zygophyllum mucronatum*, and *Z. rosowii*. All these species in the subclade I have wingless capsular fruits and stamens longer than the petals except *Z. loczyi*, which has stamens that are shorter than the petals. Subclade II consisted of *Z. macropterum, Zygophyllum neglectum, Zygophyllum pterocarpum*, and *Z. obliquum*, all of which have orange petals, stamens equal to or shorter than petals, and dark yellow seeds. Subclade III contained *Z. fabago* and *Zygophyllum macropodum*, which are morphologically united by their wingless capsules, petals that are orange-red at the base and white apically, and the stamens that are longer than the petals (Figure 8). All species of the herbaceous clade have seeds that are transparent when dry except for *Z. potaninii* and *Z. sinkiangense*.

Species of the basal grade differed from the other herbaceous species by having seeds that were opaque whether wet or dry (as opposed to transparent when dry). Moreover, between these two species, *Z. potaninii* has seeds that are dark brown with papillae, while *Z. sinkiangense* has brownish-yellow seeds with rough hairs (Figure 8). This is in contrast to the seeds of the other sampled herbaceous species, which are pale or dark yellow and have dots on the surface. The leaves *Z. potaninii* and *Z. sinkiangense* are also much bigger and fleshier than those of other species of the herbaceous clade.

All phylogenies reconstructed in this study revealed that the herbaceous species of Chinese *Zygophyllum* formed a clade, but subclades within the herbaceous group differed slightly among trees. For example, *Z. pterocarpum*, which has winged capsules, clustered with species without capsular wings (Supplementary Figures 1–4) in the phylogenetic trees from CDS, NCS, and LSC datasets but species both wide-winged and wingless capsules in the phylogeny based on complete cp genomes. Additionally, the positions of *Z. potaninii* and *Z. sinkiangense* at the base of the tree were poorly resolved. A clear intrageneric treatment of the herbaceous species will require more molecular data as well as more morphological data from fruits, seeds, flowers, and vegetative organs.

## Discussion

### Comparison of the cp Genomes of *Zygophyllum*

All sampled cp genomes of *Zygophyllum* and *Peganum* displayed a quadripartite structure that is typical for most vascular plants (Wicke et al., 2011; Tonti-Filippini et al., 2017), and the GC content was consistent with cp genomes reported for many other angiosperm taxa (Gruenstaeudl et al., 2017; Yuan et al., 2017; Mower and Vickrey, 2018). However, the complete cp genomes of *Zygophyllum* were unusually small in size compared to other closely related genera, especially *Nitraria* (159,414 bp; Abla et al., 2019), *Tetraena terrestris* (158,148 bp), *L. tridentata* (136,149 bp), *G. angustifolium* (130,809 bp), and *Peganum*, but they were somewhat similar in size to *Tetraena mongolica* (106,259 bp). Generally, the sizes of cp genomes in vascular plants range from 100 to 220 kb sequence in higher plants (Marcelo et al., 2015), so that *Zygophyllum* and *Tetraena mongolica* exhibit genomes on the small end of the range, although much smaller cp genomes have been found in nonphotosynthetic, parasitic plants, including the smallest within *Rhizanthella gardneri* (Orchidaceae) with only 59,190 bp (Delannoy et al., 2011). As with the complete cp genome of *Zygophyllum*, its IR regions (3,792–4,466 bp; Table 1) were much smaller than those of many other land plants (10–15 kb) and seed plants (20–30 kb) (Mower and Vickrey, 2018).

Beyond the genome size, the number of genes within the *Zygophyllum* cp genomes (111–112) was also generally less than observed in other land plants (Zhang et al., 2016; He et al., 2017; Hu et al., 2017; Yuan et al., 2017), aside from other closely related species sampled in this study (i.e., four species of Zygophyllaceae and two species of *Peganum*). Notably, *Zygophyllum* appears to have lost several genes within the NADH dehydrogenase complex (i.e., *ndh* genes) and undergone pseudogenization within the SC regions. This is relatively rare in plants that are not parasitic (Yu et al., 2017) but has been detected in *Najas* (Peredo et al., 2013), some species of orchids (Kim et al., 2015), Pinaceae (Braukmann et al., 2009) and gnetophytes (Wu et al., 2009). In orchids, *ndh* genes appear to have been lost several times independently, and it was speculated that this may be due to shifts between autotrophism and mycoparasitism in the family, although not all modern species exhibiting losses are mycoparasites (Kim et al., 2015). However, in Pinaceae and gnetophytes, which have no known parasitic species, the explanation is more likely that *ndh* genes became encoded in the nucleus and were subsequently lost in the cpDNA (Braukmann et al., 2009), and this could be a possibility for Z*ygophyllum*, which is also non-parasitic. The loss of several genes including *ndh* in Z*ygophyllum* may partially explain its unusually small genome size.

In addition to the loss of *ndh* genes, the IR regions of Z*ygophyllum* also lacked genes that were typically duplicated. Instead, these genes appeared in the SC regions. For example, four rRNA genes, which were commonly duplicated in the plant group, occurred only in the SSC of *Zygophyllum* as was also the case for the parasitic orchid, *R. gardneri* (Delannoy et al., 2011), which has the smallest cp genome known among land plants. Notably, we observed that the plastomes of *Peganum*, which were much larger than those of Z*ygophyllum*, contained the typically duplicated rRNA genes. Moreover, in *Peganum*, six tRNA genes were duplicated, but only three were duplicated in Z*ygophyllum*. Thus, the smaller number of genes within the IRs of Z*ygophyllum* were also likely to play a role in the genus having a relatively small plastome size.

Based on the IR boundary analysis, we found that the IRs were the most highly conserved regions within the cp genomes of *Zygophyllum* and that the IR/SC boundaries were highly similar within the genus, as is common in other plant groups (Park et al., 2017; Xu et al., 2017). However, between *Zygophyllum* and other sampled genera, the boundaries between the IRs and the SSC/LSC were considerably different. These differences likely arose due to shifts in the boundaries following the losses of genes within the IRs of *Zygophyllum*, such as losses of *ndhF* gene and rRNA genes. Nevertheless, the IR and the SSC/LSC boundaries of species of *Zygophyllum* were more similar to other Zygophyllaceae than to *P. harmala*, thus supporting the current taxonomic treatment of the latter within the related family Nitrariaceae.

We detected highly variable regions and cpSSRs within the cp genomes of 15 species of *Zygophyllum* through the sliding window analysis and by using MISA, respectively. The results of the sliding windows analysis showed that the average mutation rate of the intergenic regions within the SC regions was much higher than in the IR regions, as has been observed in other taxa (Perry and Wolfe, 2002). Higher mutation rates typically yield higher variability within intergenic spacers due to reduced selective pressures (Elspeth et al., 2005; Wang et al., 2005). Coincidentally, we detected nine highly variable regions with high Pi values in the intergenic spacer regions. Using MISA, we also found 156 cpSSRs, among which mononucleotide repeats were most abundant, and most cpSSRs were located in NCS regions. Of the mononucleotide repeats, polyA and polyT were most common within *Zygophyllum*. The prevalence of polyA and polyT cpSSRs has also been observed in other plant lineages such as *Lagerstroemia* (Zhang et al., 2020), *Primula* (Ren et al., 2018), *Fritillaria* (Bi et al., 2018), and *Allium* (Xie et al., 2019). The 9 highly variable regions and 156 cpSSRs will represent robust genetic resources for future identification and population studies of species of *Zygophyllum* in China.

### Phylogenetic and Taxonomic Implication

Our phylogenetic and comparative cp genomic results strongly supported the treatment of Zygophyllaceae as separate from Nitrariaceae, represented here by *Peganum*, (Figure 7) as in APG IV (Angiosperm Phylogeny Group (APG) IV, 2016). We also found a higher resolution for relationships between species of *Zygophyllum* in China compared to prior studies based on one or a few gene regions (Liou, 1998; Beier et al., 2003; Bellstedt et al., 2008). Notably, all well-supported ML and BI trees representing the 24 plastome sequences of *Zygophyllum* and 2 sequences of *Peganum* supported mutually monophyletic herbaceous and shrubby clades. The shrubby clade of *Z. kaschgaricum* and *Z. xanthoxylon* was sister to the herbaceous clade. The shrubby clade comprises the former species of *Sarcozygium*, which is now sometimes regarded as subgenus within *Zygophyllum*, such as the Flora of USSR (Bobrov, 1949) and which has been resolved within other, prior molecular phylogenetic studies (Beier et al., 2003; Bellstedt et al., 2008, Wu et al., 2015, 2018).

While species of the herbaceous and shrubby clades differ in several morphological characters other than their habits (i.e., herbaceous species have 10 stamens and five carpels, while shrubs have 10 stamens and three carpels), they share features, some of which distinguish them within Zygophyllaceae and support the including of the shrubby clade within the genus. For example, fruits of all species are capsules and most (except the basal grade within the herbaceous clade, *Z. potaninii* and *Z. sinkiangense*) have seeds that are transparent when wet or dry (Figure 8). Moreover, the two clades have cp genomic features that are much more similar to one another than to other genera in Zygophyllaceae (Figure 3). Prior studies have also shown that *Z. xanthoxylon* of the shrubby clade and *Z. fabago* of the herbaceous clade were both clustered with south African and Australian *Zygophyllum* (Bellstedt et al., 2008) based on molecular phylogeny, thus, supporting treatment of the shrubby species within the genus.

Within the shrubby clade, the accessions of *Z. kaschgaricum* and *Z. xanthoxylon* comprised a mixed group suggesting that they should be regarded as one species, *Z. xanthoxylon* as in the studies by Zhao and Zhu (2003) and Yang (2011). This is also consistent with our examination of morphology, in which we found more-or-less continuous variation in fruit size and shape. Neither molecular phylogeny nor morphological results supported the treatment of *Z. kaschgaricum* as a separate species as described in the study by Liou and Zhou (2008).

The herbaceous clade comprised a basal grade and three subclades. While these relationships were largely highly supported, we found that the relationships of *Z. pterocarpum, Z. potaninii*, and *Z. sinkiangense* were poorly resolved, and we observed few obvious morphological synapomorphies to support the subclades. Within the herbaceous clade, whether fruits were broad or narrow and winged or wingless, species varied broadly in the number of leaflets (4–10). Thus, resolving relationships within the herbaceous clade and establishing meaningful taxonomic boundaries within it will likely require additional morphological and molecular data.

## Conclusion

In this study, we reported the complete cp genome sequences for 24 individuals of *Zygophyllum* (Zygophyllaceae), representing 15 species distributed in China, and for 2 closely related species, *P. harmala* and *P. nigellastrum* (Nitrariaceae). Through the comparisons of these newly sequenced cp genomes to additional sequences from species of Zygophyllaceae in public databases, we found that the genomes of *Zygophyllum* were much smaller, highly conserved in gene content and order, but differed markedly from other genera within the family and from *Peganum*. Based on the comparisons with all other sampled genera, the cp genomes of *Zygophyllum* appeared to have experienced considerable gene loss, *ndh* genes, and rRNA genes. Within *Zygophyllum*, IRs were the most highly conserved regions and contained none of the nine highly variable regions and numerous SSRs that we identified. We expect that these variable regions and repeat markers, which occur within the LSC and SSC, will be useful in future phylogenetic, phylogeographic, population genetic, and genetic relationship studies on *Zygophyllum*. Our phylogenomic results based on the complete cp genomes, CDSs, LSC, and NCS supported the monophyly of *Zygophyllum* in China and its division into an herbaceous and shrubby clade. The shrubby clade is highly supported and contains two species, *Z. xanthoxylon* and *Z. kaschgaricum*, formerly regarded in the genus, *Sarcozygium*. Our phylogenetic results and comparative analyses of cp genomic structures supported the treatment of *Sarcozygium* in *Zygophyllum*. The sampled accessions of the two members of the shrubby clade did not form mutually monophyletic groups. Therefore, based on this and morphological evidence, we recommend that *Z. kaschgaricum* be merged into *Z. xanthoxylon*. Overall, these results demonstrated the power of cp plastomes to improve the phylogenetic resolution of species and to resolve taxonomic questions.

## Data Availability Statement

The original contributions presented in the study are publicly available. This data can be found here: accession numbers in Table 2.

## Data Accessibility

All the sequences were deposited as GenBank: Accessions MW307829, MW387262–MW387266, MW417249–MK417256, MW477239–MW477240, MW489448–MW489449, MW525443–MW525444, MW551563–MW551564, MW557318–MW557319, MW771516–MW771517.

## Author Contributions

LD, Z-YC, and LZ designed the study and collected all samples. LZ, SW, and CS led data analysis and the making of figures and tables with assistance from J-RW, NS, and LZ. The manuscript was drafted by LZ, AH, LD, and Z-YC edited the manuscript for structure, language, and scientific content. All authors approved the final manuscript.

## Funding

This study was supported by the National Natural Science Foundation of China (Grant No. 31660048 and 32070229).

## Conflict of Interest

The authors declare that the research was conducted in the absence of any commercial or financial relationships that could be construed as a potential conflict of interest.

## Publisher's Note

All claims expressed in this article are solely those of the authors and do not necessarily represent those of their affiliated organizations, or those of the publisher, the editors and the reviewers. Any product that may be evaluated in this article, or claim that may be made by its manufacturer, is not guaranteed or endorsed by the publisher.

## Supplementary Material

Supplementary Figure 1 Phylogenetic tree constructed using Maximum Likelihood (ML) and Bayesian Inference (BI) methods, based on the CDS sequences from different species.

Supplementary Figure 2 Phylogenetic tree constructed using Maximum Likelihood (ML) based on the NCS sequences from different species.

Supplementary Figure 3 Phylogenetic tree constructed using Bayesian Inference (BI) methods, based on the NCS sequences from different species.

Supplementary Figure 4 Phylogenetic tree constructed using Maximum Likelihood (ML) and Bayesian Inference (BI) methods, based on the LSC sequences from different species.

Supplementary Figure 5 Phylogenetic tree constructed using Maximum Likelihood (ML) based on the SSC sequences from different species.

Supplementary Figure 6 Phylogenetic tree constructed using Maximum Likelihood (ML) IR sequences from different species.

Supplementary Table 1 Information of reported chloroplast genomes in outgroup species.

Supplementary Table 2 The number, type, and distribution of SSRs of 24 complete cp genomes in *Zygophyllum*.

Supplementary Table 3 The size and location of SSRs from 24 complete cp genomes in *Zygophyllum*.
