# Supplementary figures and images for: Comparative Chloroplast Genomics and Phylogenetic Analysis of Zygophyllum (Zygophyllaceae) of China
Source: Front Plant Sci. 2021 Sep 24;12:723622. doi: 10.3389/fpls.2021.723622 (PMC8500179; doi:10.3389/fpls.2021.723622)

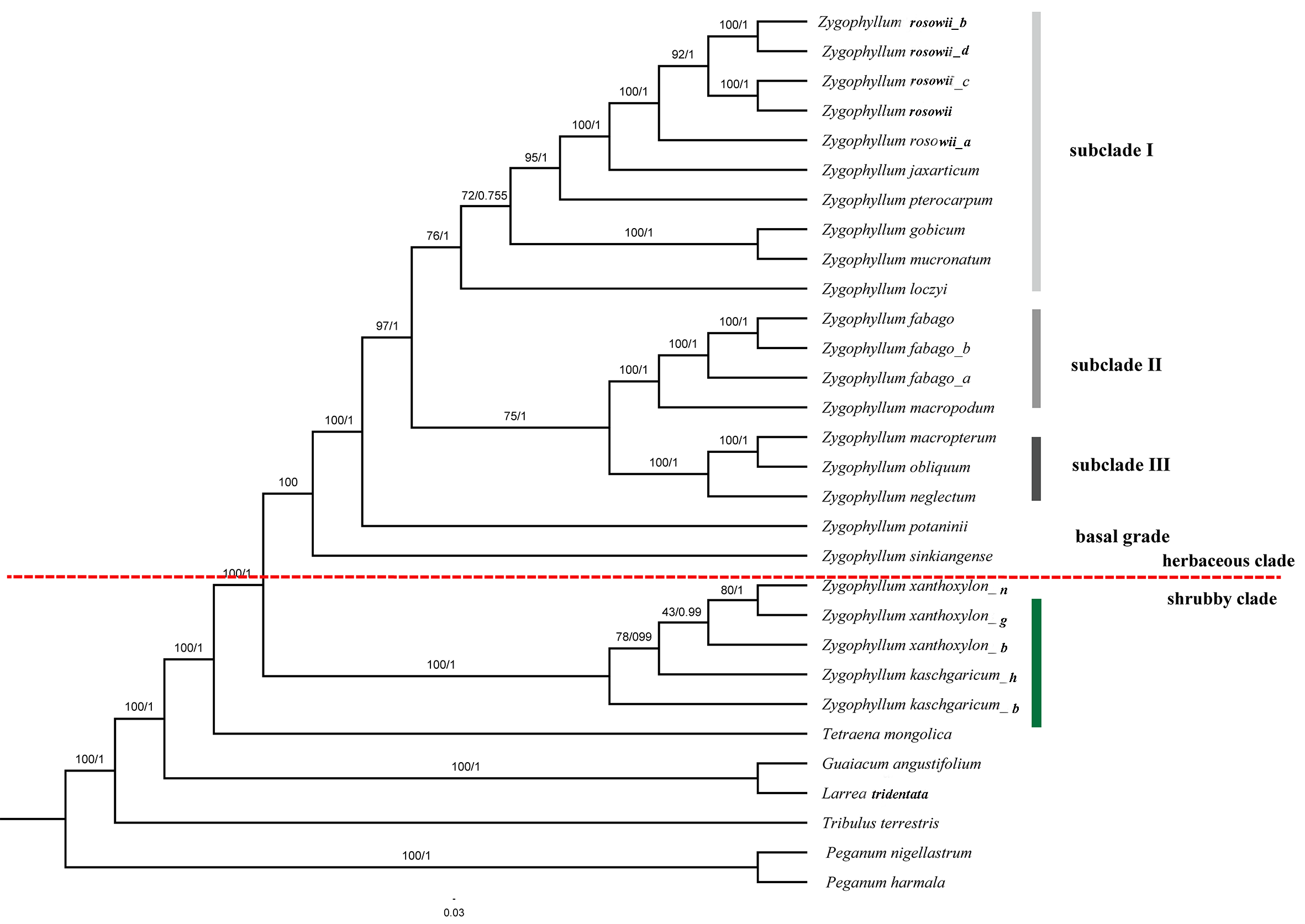

Supplement: Supplementary Figure 1 — Phylogenetic tree constructed using Maximum Likelihood (ML) and Bayesian Inference (BI) methods, based on the CDS sequences from different species. [file Data_Sheet_2.ZIP › Figure S1 Phylogenetic tree constructed using Maximum Likelihood (ML) and Bayesian Inference (BI) methods, based on the CDS sequences from different species.tif]

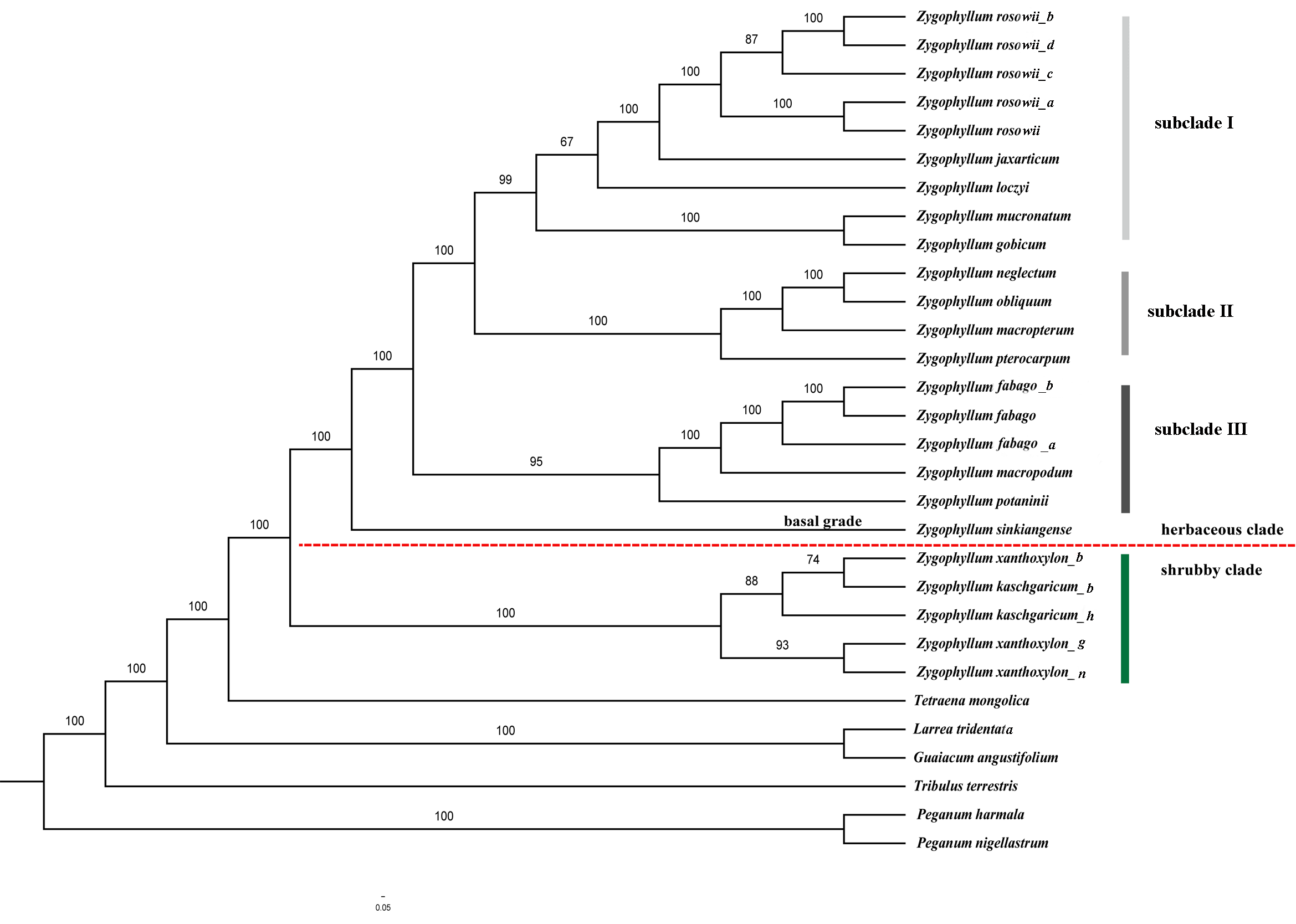

Supplement: Supplementary Figure 1 — Phylogenetic tree constructed using Maximum Likelihood (ML) and Bayesian Inference (BI) methods, based on the CDS sequences from different species. [file Data_Sheet_2.ZIP › Figure S2 Phylogenetic tree constructed using Maximum Likelihood (ML) based on the NCS sequences from different species.tif]

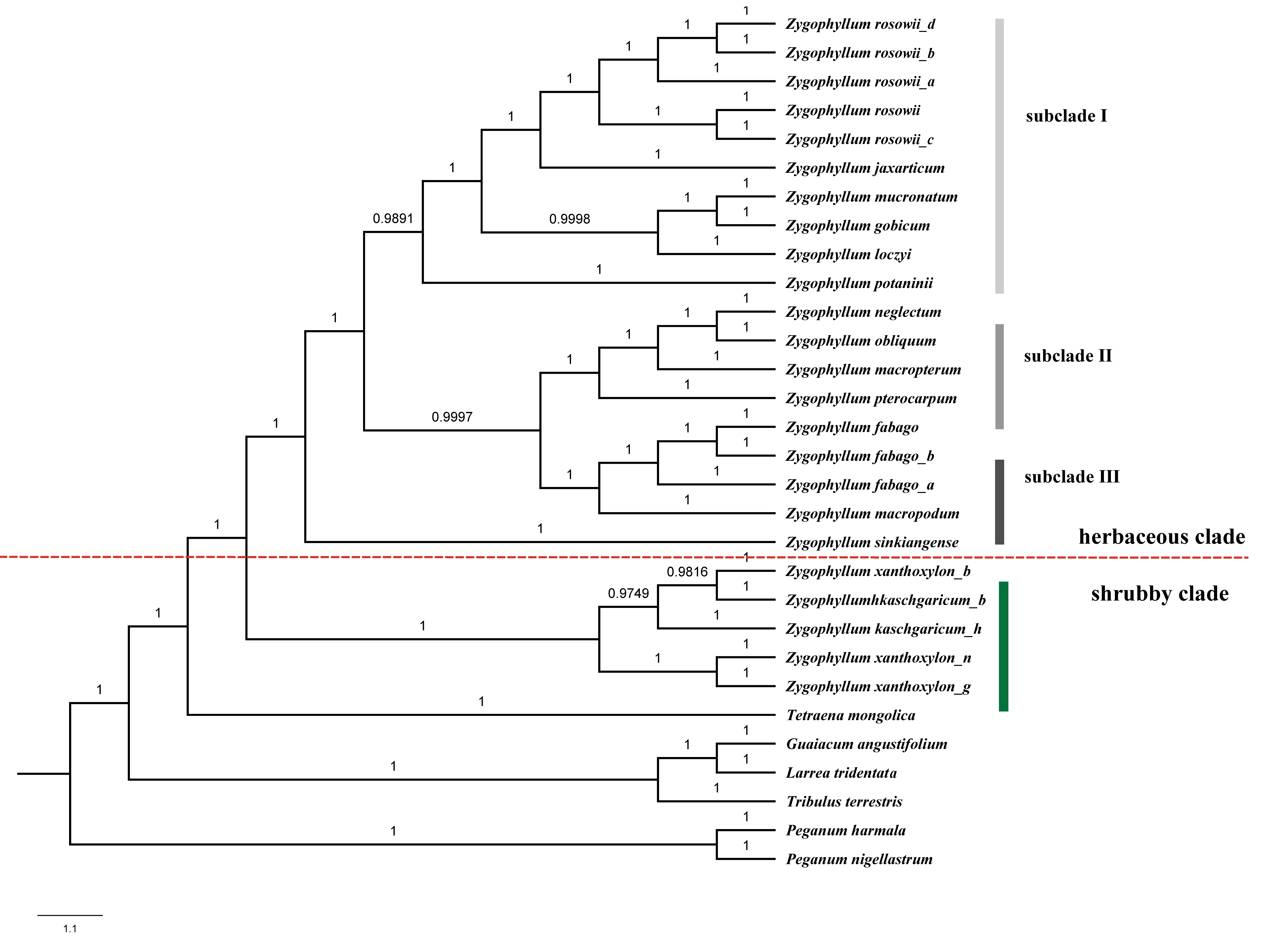

Supplement: Supplementary Figure 1 — Phylogenetic tree constructed using Maximum Likelihood (ML) and Bayesian Inference (BI) methods, based on the CDS sequences from different species. [file Data_Sheet_2.ZIP › Figure S3 Phylogenetic tree constructed using Bayesian Inference (BI) methods, based on the NCS sequences from different species.tif]

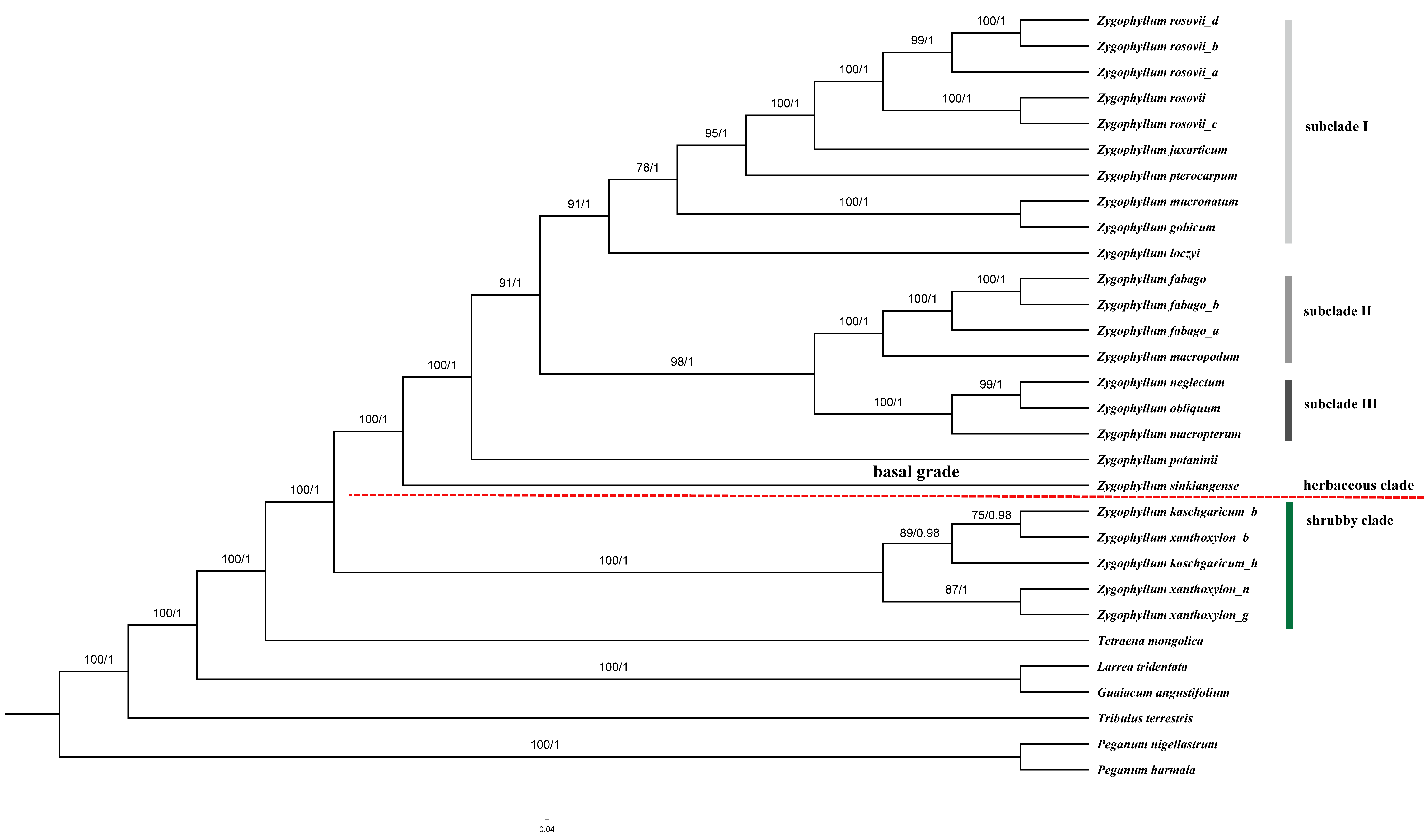

Supplement: Supplementary Figure 1 — Phylogenetic tree constructed using Maximum Likelihood (ML) and Bayesian Inference (BI) methods, based on the CDS sequences from different species. [file Data_Sheet_2.ZIP › Figure S4 Phylogenetic tree constructed using Maximum Likelihood (ML) and Bayesian Inference (BI) methods, based on the LSC sequences from different species.tif]

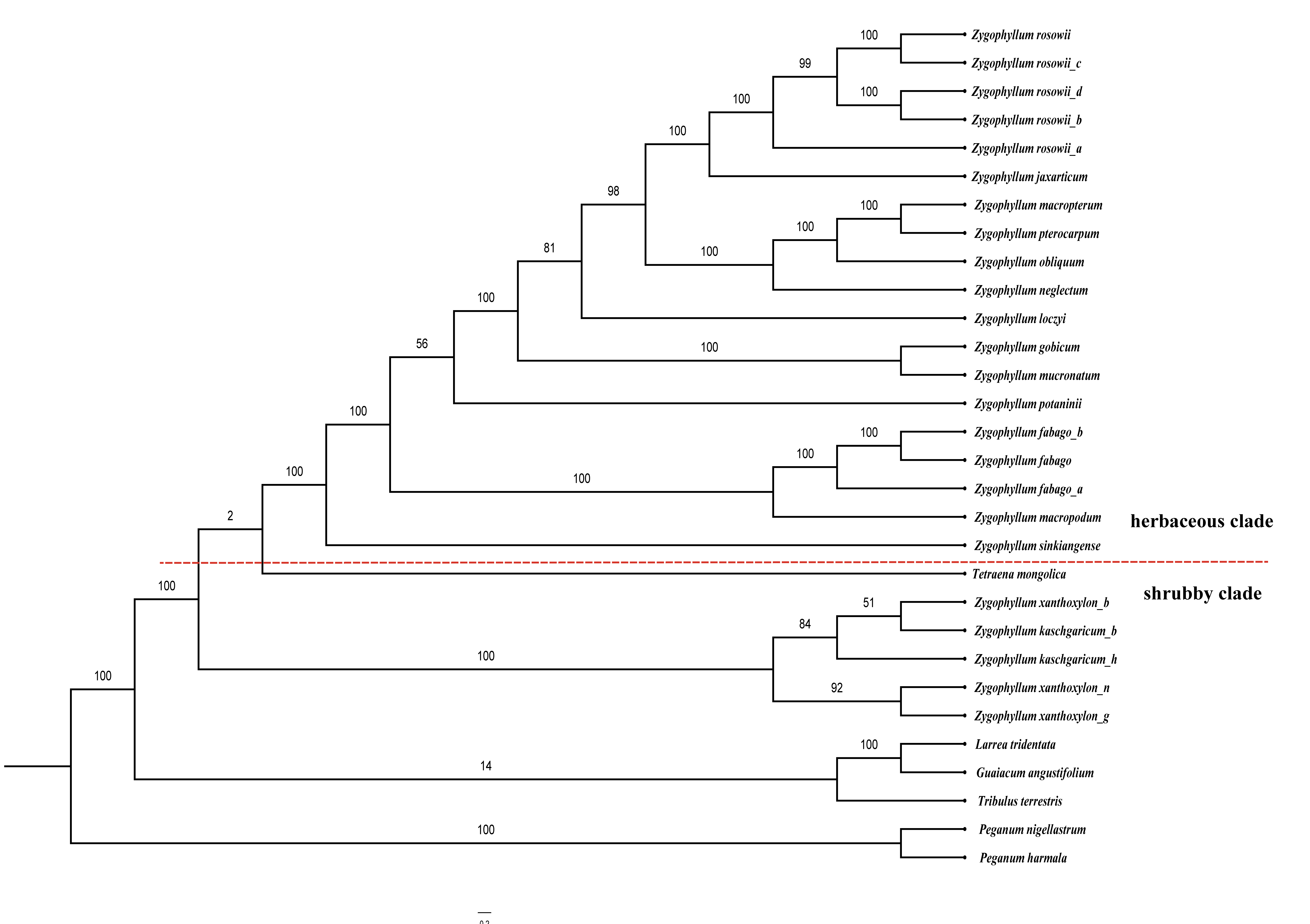

Supplement: Supplementary Figure 1 — Phylogenetic tree constructed using Maximum Likelihood (ML) and Bayesian Inference (BI) methods, based on the CDS sequences from different species. [file Data_Sheet_2.ZIP › Figure S5 Phylogenetic tree constructed using Maximum Likelihood (ML) based on the SSC sequences from different species .tif]

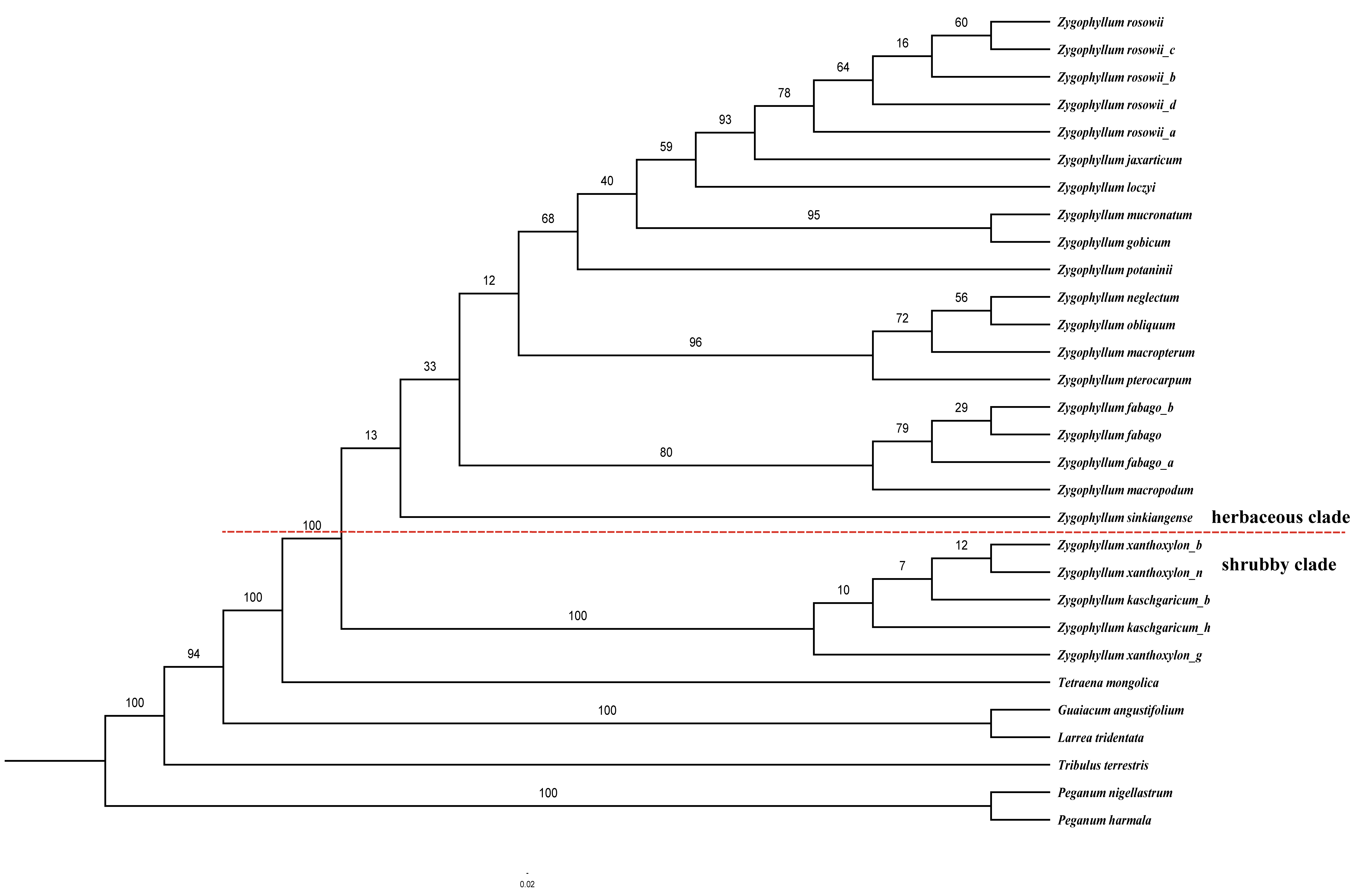

Supplement: Supplementary Figure 1 — Phylogenetic tree constructed using Maximum Likelihood (ML) and Bayesian Inference (BI) methods, based on the CDS sequences from different species. [file Data_Sheet_2.ZIP › Figure S6 Phylogenetic tree constructed using Maximum Likelihood (ML) IR sequences from different species.tif]
