# Supplementary material for: Comparative Chloroplast Genomics and Phylogenetic Analysis of Zygophyllum (Zygophyllaceae) of China
Source: Front Plant Sci. 2021 Sep 24;12:723622. doi: 10.3389/fpls.2021.723622 (PMC8500179; doi:10.3389/fpls.2021.723622)
Supplement: Supplementary Table 1 — Information of reported chloroplast genomes in outgroup species. [file Data_Sheet_1.ZIP › Table S1.docx]

**Table S1.** Information of reported chloroplast genomes in outgroup species

| **Organism Name** | **Size(bp)** | **GC%** | **Type** | **Replicons** |
| --- | --- | --- | --- | --- |
| *Tetraena mongolica* | 106,259 | 33.6 | chloroplast | MH325021.1 |
| *Larrea tridentate* | 136,194 | 35.1 | chloroplast | KT272174.1 |
| *Guaicum angustifolium* | 130,809 | 35.6 | chloroplast | MK726011.1 |
| *Tribulus terrestris* | 158,184 | 35.8 | chloroplast | NC_046758 |
